# Targeting Oxidative Stress with Antioxidant Duotherapy after Experimental Traumatic Brain Injury

**DOI:** 10.3390/ijms221910555

**Published:** 2021-09-29

**Authors:** Jenni Kyyriäinen, Natallie Kajevu, Ivette Bañuelos, Leonardo Lara, Anssi Lipponen, Silvia Balosso, Elina Hämäläinen, Shalini Das Gupta, Noora Puhakka, Teemu Natunen, Teresa Ravizza, Annamaria Vezzani, Mikko Hiltunen, Asla Pitkänen

**Affiliations:** 1 A. I. Virtanen Institute for Molecular Sciences, University of Eastern Finland, FI-70211 Kuopio, Finland; jenni.kyyriainen@uef.fi (J.K.); natallie.kajevu@uef.fi (N.K.); ivette.banuelos-cabrera@uef.fi (I.B.); Leonardo.laravalderrabano@uef.fi (L.L.); anssi.lipponen@uef.fi (A.L.); elina.hamalainen@uef.fi (E.H.); shalini.gupta@uef.fi (S.D.G.); noora.puhakka@uef.fi (N.P.); 2 Department of Health Security, Finnish Institute for Health and Welfare, FI-70701 Kuopio, Finland; 3 Department of Neuroscience, Mario Negri Institute for Pharmacological Research IRCCS, 20156 Milano, Italy; silvia.balosso@marionegri.it (S.B.); teresa.ravizza@marionegri.it (T.R.); annamaria.vezzani@marionegri.it (A.V.); 4 Institute of Biomedicine, University of Eastern Finland, FI-70211 Kuopio, Finland; teemu.natunen@uef.fi (T.N.); mikko.hiltunen@uef.fi (M.H.)

**Keywords:** antioxidant treatment, cytokine, lateral fluid-percussion injury, *N*-acetylcysteine, oxidative stress, sulforaphane

## Abstract

We assessed the effect of antioxidant therapy using the Food and Drug Administration-approved respiratory drug *N*-acetylcysteine (NAC) or sulforaphane (SFN) as monotherapies or duotherapy in vitro in neuron-BV2 microglial co-cultures and validated the results in a lateral fluid-percussion model of TBI in rats. As in vitro measures, we assessed neuronal viability by microtubule-associated-protein 2 immunostaining, neuroinflammation by monitoring tumor necrosis factor (TNF) levels, and neurotoxicity by measuring nitrite levels. In vitro, duotherapy with NAC and SFN reduced nitrite levels to 40% (*p* < 0.001) and neuroinflammation to –29% (*p* < 0.001) compared with untreated culture. The treatment also improved neuronal viability up to 72% of that in a positive control (*p* < 0.001). The effect of NAC was negligible, however, compared with SFN. In vivo, antioxidant duotherapy slightly improved performance in the beam walking test. Interestingly, duotherapy treatment decreased the plasma interleukin-6 and TNF levels in sham-operated controls (*p* < 0.05). After TBI, no treatment effect on HMGB1 or plasma cytokine levels was detected. Also, no treatment effects on the composite neuroscore or cortical lesion area were detected. The robust favorable effect of duotherapy on neuroprotection, neuroinflammation, and oxidative stress in neuron-BV2 microglial co-cultures translated to modest favorable in vivo effects in a severe TBI model.

## 1. Introduction

Traumatic brain injury (TBI) is defined as an alteration in brain function or other evidence of brain pathology caused by an external force [1]. Annually, approximately 2.5 million people in both Europe [2] and the United States [3] experience TBI. Depending on the location, type, and severity, TBI can result in different types of cognitive, emotional, and behavioral comorbidities [4,5].

Attempts to improve the TBI outcome are focused on mitigating the molecular cascades that lead to worsening of the secondary injury and/or on enhancing recovery [6,7]. Secondary injury comprises multifaceted temporally orchestrated pathologies, including apoptosis, neuroinflammation, and generation of reactive oxygen species (ROS) that lead to oxidative stress, and blood–brain barrier (BBB) dysfunction [8,9,10]. Therapeutic approaches tackling more than one molecular pathology as they appear may be needed to optimize the functional gain [11].

One objective of the European Framework 7-funded consortium EPITARGET [12] is to identify monotherapies and polytherapies that alleviate pathologies common to different etiologies causing structural epilepsies such as status epilepticus (SE) and TBI. Recently, Pauletti et al., [13] demonstrated that N-acetylcysteine (NAC) and sulforaphane (SFN) duotherapy has disease-modifying neuroprotective, antioxidant, and anti-inflammatory effects by mitigating the SE-induced epileptogenesis and pathologic outcome. 

Monotherapy with NAC, a precursor of glutathione, has beneficial effects on the pathologic and functional outcome after TBI. Previous studies demonstrated neuroprotective effects of NAC monotherapy in the cortex, hippocampus, and thalamus in a lateral fluid-percussion injury (FPI) model at 24 h post-injury [14,15]. Administration of NAC within 15 to 60 min after injury decreased apoptosis and inflammatory markers such as interleukin (IL)-1β or tumor necrosis factor (TNF) in a weight drop model assessed at 3 d post-TBI [16,17]. NAC also increased glutathione levels and decreased ROS levels in the hippocampus at 3 d after weight-drop [17,18] or at 12 h after controlled cortical impact (CCI)-induced TBI [19]. Interestingly, oral NAC treatment started within 24 h post-injury alleviated various symptoms in humans with combat blast-related mild TBI sequelae such as headache, confusion, and abnormal sleep [20]. 

SFN activates transcription factor NF-E2 p45-related factor 2 (Nrf2)-dependent transcription of detoxification enzymes [21,22]. Nrf2 protein, encoded by the *Nfe2l2* gene, regulates the expression of genes involved in natural antioxidant and anti-inflammatory defense [23,24]. In addition, Nrf2 regulates antioxidant target genes such as NAD(P)H quinone oxidoreductase 1 (*Nqo1*), heme oxygenase 1 (*Hmox1*), and glutamate cysteine ligase modifier (*Gclm*), which inhibit transcription of proinflammatory mediators [25]. Previous in vitro studies in immature hippocampal neurons revealed neuroprotective effects of SFN against cell death induced by oxygen and glucose deprivation [26]. After CCI in rats, SFN decreased oxidative damage and reduced the number of dying neurons at 24 h, leading to reduced lesion volume at 7 d post-injury [27].

Our objective was to investigate whether the favorable disease-modifying effects of NAC+SFN duotherapy described by Pauletti et al., [13] in an SE model could be expanded to a TBI model. In particular, we assessed whether NAC+SFN duotherapy continued over the acute and sub-acute post-injury period mitigated post-TBI inflammatory status and improved functional outcome. 

## 2. Results

**Figure 1:**
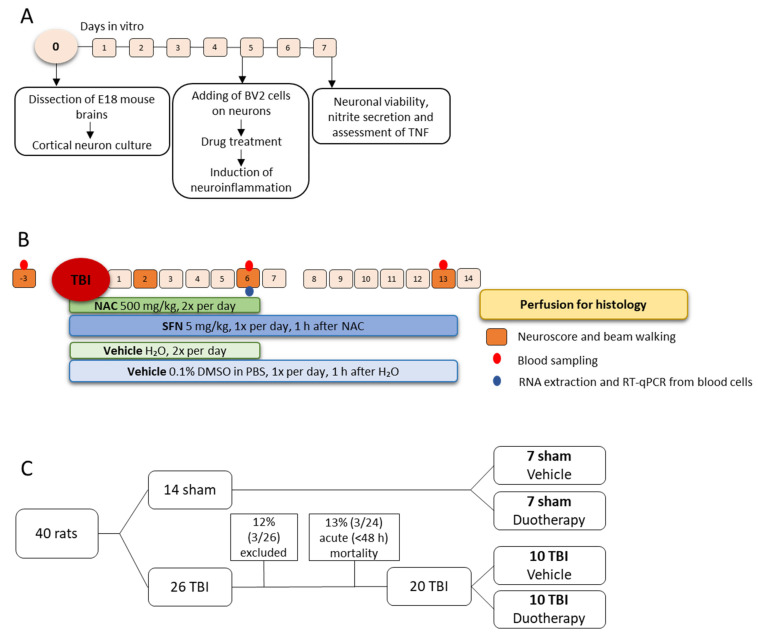
Study design and randomization. (**A**) In vitro study design. Primary cortical neuronal culture was prepared from E18 mouse brains. At 5 d in vitro (DIV), BV2-microglia cells were added to the culture and cells were pre-treated with N-acetylcysteine (NAC) or sulforaphane (SFN) monotherapy or SFN+NAC duotherapy under inflammatory conditions. Neuronal viability was assessed using microtubule-associated-protein 2 immunostaining described by [28]. Finally, secreted nitrite and TNF concentrations in the cell culture medium were assessed using Griess reagent and ELISA, respectively. (**B**) In vivo study design. Sham-operated experimental controls and rats with traumatic brain injury (TBI) induced with lateral fluid-percussion injury were followed-up for 2 weeks. Administration of vehicle (sterile H_2_O) or NAC (500 mg/kg in sterile H_2_O, i.p.) was started at 1 h post-impact. At 1 h later (*i.e.*, at 2 h after TBI or sham-operation), rats were treated with vehicle (0.1% DMSO in PBS, pH 7.4, i.p.) or SFN (5 mg/kg in 0.1% DMSO in PBS, i.p.). NAC was administered twice a day (6 h apart) and SFN was administered once a day (between NAC injections). Neuroscore and beam walking tests were performed at baseline, and thereafter on days (D) 2, D6, and D13 post-TBI (injury on D0). Body weight was measured at D0, and then daily until the rats were killed. Tail vein blood was sampled for biomarker analysis at baseline, and on D6 and D13 post-TBI. On D14, rats were transcardially perfused for histology. (**C**) Randomization. A total 40 rats were included into the study. Of these, 14 animals were randomized to sham-operation and 26 to lateral fluid-percussion-induced TBI, and further, to vehicle or duotherapy treatments. One rat in the TBI group was excluded due to broken dura after the craniectomy, another rat was excluded due to a disconnected injury cap, and another due to broken dura after the impact. Acute post-impact mortality was 13%. Thus, there were 7 rats in the Sham-VEH, 7 in Sham-DUO, 10 in TBI-VEH, and 10 in TBI-DUO groups. Abbreviations: D, day; DIV, days in vitro; DMSO, dimethyl sulfoxide; DUO, duotherapy treated with N-acetylcysteine and sulforaphane; ELISA, enzyme-linked immunosorbent assay; h, hour; i.p.; intraperitoneal; NAC, N-acetylcysteine; PBS, phosphate buffered saline; RNA, ribonucleic acid; RT-qPCR, reverse transcription quantitative polymerase chain reaction; SFN, sulforaphane; TBI, traumatic brain injury; TNF, tumor necrosis factor; VEH, vehicle; wk, week.

Study design of in vitro and in vivo experiments is summarized in Figure 1.

### 2.1. Effect of Treatments on In Vitro Response Biomarkers

Co-cultures were treated with four different concentrations of NAC, SFN, or SFN+NAC before induction of neuroinflammation. The levels of response biomarkers (see below) in the medium of treated lipopolysaccharide (LPS)/ interferon (INF)γ+ -exposed cultures were compared with those of untreated LPS/INFγ+ -exposed cultures.

#### 2.1.1. Neuronal Viability

**Figure 2:**
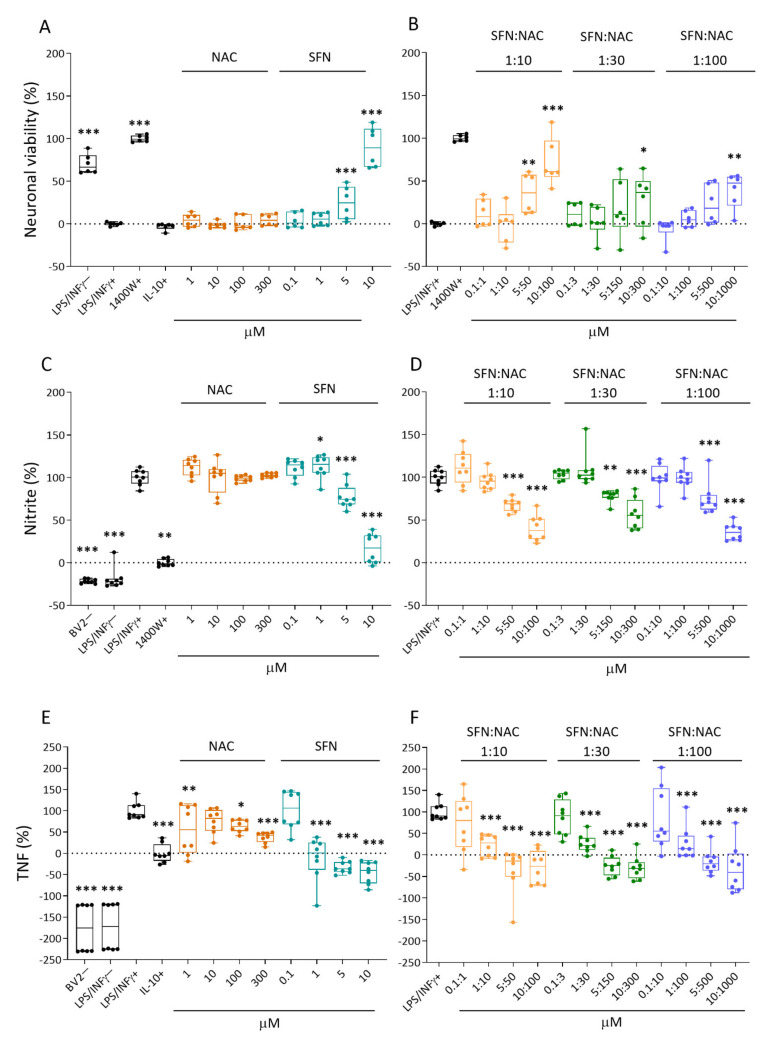
Effect of NAC or SFN monotherapy and SFN+NAC duotherapy on neuronal viability, and nitrite and TNF levels in cortical neuron-BV2 co-cultures in vitro. (**A**) Monotherapy with NAC did not affect the LPS/INFγ+ -induced cell death (*p* > 0.05). SFN monotherapy at 5 µM (*p* < 0.001) and 10 µM (*p* < 0.001) concentrations increased cell survival, a dose of 10 µM being almost as effective as the iNOS inhibitor 1400W (positive treatment control). Note that the anti-inflammatory cytokine IL-10 did not increase cell survival. (**B**) The 2 highest concentrations of 1:10 SFN+NAC duotherapy ratios (5 µM+50 µM [*p* < 0.001] and 10 µM+100 µM [*p* < 0.001]) promoted neuronal survival. The effect of the highest concentrations at ratios of 1:30 and 1:100 (10 µM+300 µM [*p* < 0.05]; 10 µM+1000 µM [*p* < 0.01]) were meager. Note that in panels A and B neuronal viability was less in those particular wells with value below 0% than on average in the LPS/INFγ+ -treated wells. Furthermore, the compound was better in those particular wells with value above 100% than the positive control. (**C**) Monotherapy with NAC did not affect the LPS/INFγ+ -induced increase in nitrite levels in the cell culture medium (*p* > 0.05 compared with LPS/INFγ+ ₋treated cultures [100%], to which the nitric oxide levels in all other samples were normalized). SFN induced a dose-dependent reduction in nitrite levels, a dose of 10 µM (*p* < 0.001) being almost as effective as the iNOS inhibitor 1400W (inhibitor of iNOS, positive treatment control). Note that the BV2− (no BV2 cells, only cortical neuronal cells) and LPS/INFγ− cultures were not exposed to a neuroinflammatory agent (LPS/INFγ+), and thus showed no nitrite release into the culture medium (baseline). The positive treatment control, 1400W, reduced the nitrite levels almost to the baseline level. (**D**) The 2 highest concentrations of each of the 3 SFN+NAC duotherapy ratios reduced the LPS/INFγ+ -induced increase in nitrite levels in a dose-dependent manner. The overall SFN+NAC duotherapy effect on nitrite levels was comparable to that of SFN monotherapy. (**E**) Monotherapy both with NAC and SFN reduced the LPS/INFγ+ -induced increase in TNF levels in a dose-dependent manner. The SFN monotherapy at 5 µM (*p* < 0.001) or 10 µM (*p* < 0.001) concentrations reduced the TNF levels even more than treatment with the positive control, IL-10 (TNF inhibitor). (**F**) All 3 SFN+NAC duotherapy ratios reduced the LPS/INFγ+ -induced increase in TNF levels in a dose-dependent manner. Note that in panels C to F nitrite and TNF levels were higher in those particular wells with value above 100% than on average in the LPS/INFγ+ -treated wells. Furthermore, the compound was better in those particular wells with value below 0% than the positive control. Abbreviations: IL-10, interleukin 10; INFγ, Interferon gamma; LPS, lipopolysaccharide; NAC, N-acetylcysteine; SFN, sulforaphane; TNF, tumor necrosis factor. Statistical significances: * *p* < 0.05, ** *p* < 0.01 and *** *p* < 0.001 compared to the untreated co-culture exposed to LPS/INFγ+. In A-D n = 8 experimental repeats (2 batches, 4 wells/batch) and E-F n = 6 repeats (2 batches, 3 wells/batch). Data were analyzed using linear regression models in R and presented as whisker-plots with mean.

Neuronal viability in untreated LPS/INFγ+ -exposed co-culture was set to 0%. Neuronal viability in a culture treated with a positive control, inducible nitric oxide (iNOS) inhibitor 1400W, was set to 100% (*p* < 0.001). Efficacy of the test drugs is expressed as a percentage of that in the 1400W-treated LPS/INFγ+ culture. Data are summarized in Figure 2A–B.

Controls. The negative control culture (LPS/INFγ−) showed very little neuronal damage with 70% neuronal viability (*p* < 0.001). IL-10 had no effect on neuronal viability (–3%, *p* > 0.05) (Figure 2A). 

Monotherapy. NAC did not improve neuronal viability at any concentration evaluated (all *p* > 0.05). SFN increased neuronal viability to 25% that in the positive control at 5 µM (*p* < 0.001) and to 90% at 10 µM (*p* < 0.001) (Figure 2A).

Duotherapy. SFN+NAC duotherapy improved neuronal survival in a dose-dependent manner at the 2 highest concentrations (Figure 2B). At a ratio of 1:10, SFN:NAC concentrations of 5 µM:50 µM increased neuronal viability to 36% (*p* < 0.01) and 10 µM:100 µM concentrations increased neuronal viability to 72% of that in the positive control (*p* < 0.001). At a ratio of 1:30, SFN:NAC concentrations of 10 µM:300 µM increased neuronal viability to 28% (*p* < 0.05). Finally, at a ratio of 1:100, SFN:NAC concentrations of 10 µM:1000 µM increased neuronal viability to 39% (*p* < 0.01).

#### 2.1.2. Nitrite Levels

Nitrite levels released into the culture medium were measured as a response biomarker for nitric oxide-mediated neurotoxicity.

The nitrite level in the culture medium of untreated LPS/INFγ+ -exposed co-culture was set to 100%. The nitrite level in the culture after treatment with the iNOS inhibitor 1400W was set to 0% (*p* < 0.01). The efficacy of the test drugs was expressed as a percentage of nitrite levels in the culture medium compared with that in the untreated culture (LPS/INFγ+). Data are summarized in Figure 2C,D.

Controls. As expected, medium from the negative control cultures had nitrite levels that were even lower than those in 1400W-treated cultures (BV2− –22%, *p* < 0.001; LPS/INFγ− –19%, *p* < 0.001) (Figure 2C).

Monotherapy. NAC did not affect the nitrite levels at any concentration evaluated (all, *p* > 0.05). SFN induced a dose-dependent reduction in nitrite levels. At concentration of 5 µM, SFN reduced nitrite levels to 77% (*p* < 0.001) and at 10 µM, 17% (*p* < 0.001) (Figure 2C).

Duotherapy. SFN+NAC duotherapy decreased the nitrite levels in a dose-dependent manner at the 2 highest concentrations (Figure 2D). At a ratio of 1:10, SFN:NAC concentrations of 5 µM:50 µM reduced nitrite levels to 68% (*p* < 0.001) and concentrations of 10 µM:100 µM reduced nitrite levels to 40% (*p* < 0.001). At a ratio of 1:30, SFN:NAC concentrations of 5 µM:150 µM reduced nitrite levels to 78% (*p* < 0.01) and 10 µM:300 µM reduced nitrite levels to 57% (*p* < 0.001). Finally, at a ratio of 1:100, SFN:NAC concentrations of 5 µM:500 µM reduced nitrite levels to 75% (*p* < 0.001) and 10 µM:1000 µM reduced nitrite levels to 36%, (*p* < 0.001). 

#### 2.1.3. Tumor Necrosis Factor (TNF) Secretion

TNF levels released into the culture medium were measured as a response biomarker for neuroinflammation.

TNF levels in the culture medium from untreated LPS/INFγ+ -exposed co-culture was set to 100%. TNF levels in the culture after treatment with IL-10 was set to 0% (*p* < 0.001). Efficacy of the test drugs was expressed as a percentage of TNF in the culture medium as compared with that in untreated culture (LPS/INFγ+). Data are summarized in Figure 2E,F.

Controls. Negative control cultures showed low levels of TNF, even lower than in IL10-treated cultures (BV2− –176%, *p* < 0.001; LPS/INFγ− –172%, *p* < 0.001) (Figure 2E). 

Monotherapy. NAC at concentrations of 1, 100, and 300 µM reduced TNF levels to 56% (*p* < 0.01), 64% (*p* < 0.05), and 38% (*p* < 0.001), respectively. SFN at concentrations of 1, 5, and 10 µM decreased TNF levels dose-dependently to –12% (*p* < 0.001), –33% (*p* < 0.001), and –46% (*p* < 0.001), respectively (Figure 2E). 

Duotherapy. SFN:NAC duotherapy decreased the TNF levels in a dose-dependent manner at the 3 highest concentrations (Figure 2F). At a ratio of 1:10, SFN:NAC concentrations of 1 µM:10 µM, 5 µM:50 µM, and 10 µM:100 µM decreased TNF levels to 22% (*p* < 0.001), –35% (*p* < 0.001), and –29% (*p* < 0.001), respectively. At a ratio of 1:30; SFN:NAC concentrations of 1 µM:30 µM, 5 µM:150 µM, and 10 µM:300 µM decreased TNF levels to 26% (*p* < 0.001), 25% (*p* < 0.001), and –30% (*p* < 0.001), respectively. At a ratio of 1:100, SFN:NAC concentrations of 1 µM:100 µM, 5 µM:500 µM, and 10 µM:1000 µM decreased TNF levels to 27% (*p* < 0.001), –15% (*p* < 0.001), and –32% (*p* < 0.001), respectively.

### 2.2. In Vivo Efficacy on TBI Outcome

#### 2.2.1. Acute Post-Impact Mortality, Exclusions, Apnea Time, Time to Righting, Occurrence of Acute Seizure-like Behavior and Weight

Mortality and exclusions. Of the 40 rats, 14 were randomized to sham and 26 to TBI groups. Of the 14 sham-operated experimental controls, 7 were randomized to the Sham-VEH and 7 to Sham-DUO groups. Of the 26 rats in the TBI group, 3 rats were excluded: 1 due to a broken dura after craniectomy (1/26, 4%), 1 due to disconnected injury cap (1/26, 4%), and 1 due to a broken dura after the impact (1/26, 4%). Acute impact-related mortality was 12% (2 of the 24 injured animals). Thus, treatment was started in 21 rats with TBI (11 TBI-VEH, 10 TBI-DUO). One TBI-VEH rat was found dead at 1 d post-TBI (1/21, 5%). Altogether, 20 TBI rats completed the treatment (10 TBI-VEH, 10 TBI-DUO).

**Figure 3:**
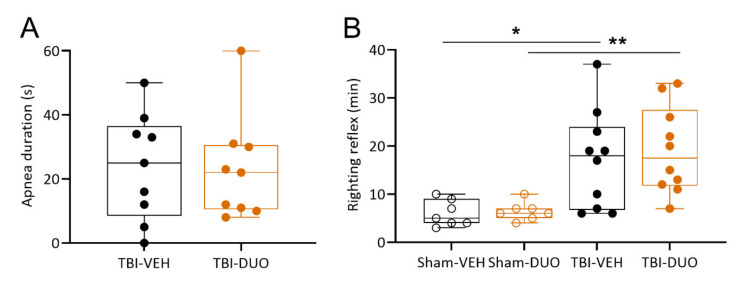
Apnea duration (s) and time to righting (min) in rats randomized to different treatment groups. (**A**) Apnea duration did not differ between rats with TBI randomized to either vehicle (TBI-VEH) or NAC+SFN duotherapy (TBI-DUO) (*p* > 0.05, independent sample t-test). (**B**) Time to righting was longer in rats with TBI than in sham-operated experimental controls (TBI-VEH vs. Sham-VEH, *p* < 0.05; TBI-DUO vs. Sham-DUO, *p* < 0.01). No difference was detected between rats randomized to the TBI-VEH or TBI-DUO groups (*p* > 0.05, independent sample *t*-test). Data are presented as whisker-plots with mean and minimum/maximum. Abbreviations: D, day; DUO, duotherapy treated with N-acetylcysteine and sulforaphane; NAC, N-acetylcysteine; SFN, sulforaphane; TBI, traumatic brain injury; VEH, vehicle. Statistical significances: * *p* < 0.05, between Sham-VEH and TBI-VEH groups (independent sample t-test), ** *p* < 0.01 between Sham-DUO and TBI-DUO groups (independent sample t-test).

Apnea. An independent sample *t*-test indicated no difference in post-impact apnea times (s) between groups (TBI-VEH: 24 ± 6 s vs. TBI-DUO: 23 ± 6 s, *p* > 0.05, Figure 3). 

Righting reflex. Similarly, no difference was detected in the time to righting (min) between the Sham-VEH and Sham-DUO groups (6.0 ± 1.0 min vs. 6.4 ± 0.7 min, *p* > 0.05) or between the TBI-VEH and TBI-DUO groups (17.1 ± 3.2 min vs. 19.1 ± 2.9 min, *p* > 0.05) (Figure 3). As expected, time to righting was delayed in TBI rats compared with sham-operated experimental controls (TBI 18.1 ± 2.1 min vs. Sham 6.2 ± 2.3 min, *p* < 0.001).

Acute post-impact seizure-like behavior. Acute post-impact seizure-like behavior was observed in 2 TBI rats; 1 of the 2 rats was randomized to the TBI-DUO group and the other rat died before the righting reflex was evaluated.

**Figure 4:**
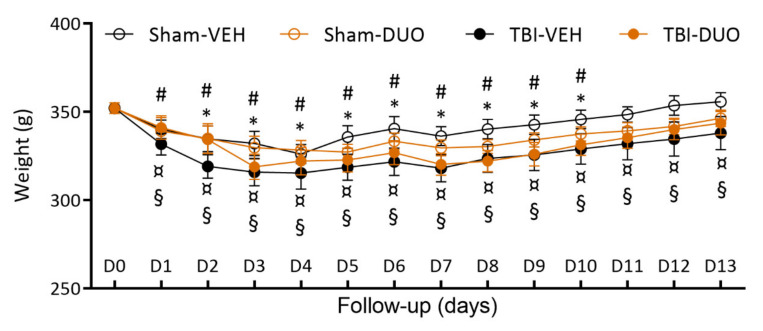
Body weight. Mean body weight of each treatment group at different time-points after sham-operation or traumatic brain injury (TBI) over the 2-week follow-up. The weight of Sham-VEH or Sham-DUO treated animals returned to the pre-operation (day [D] 0) weight by D11. TBI-VEH or TBI-DUO treated animals, although they gained weight, did not reach their D0 weight during the follow-up. Overall, body weight did not differ between treatment groups at any time-point (*p* > 0.05, Mann–Whitney *U*-test). Abbreviations: D, day; DUO, duotherapy treated with N-acetylcysteine and sulforaphane; g, gram; TBI, traumatic brain injury; VEH, vehicle; wk, week. Statistical significances: * *p* < 0.05, compared to D0 within Sham-VEH group; # *p* < 0.05, compared to D0 within Sham-DUO group; ¤ *p* < 0.05, compared to D0 within TBI-VEH group; § *p* < 0.05, compared to D0 within TBI-DUO group (Wilcoxon). Data are presented as mean ± SEM.

Weight. Mean weight was reduced on average by 5% in Sham-VEH, 6% in Sham-DUO, 9% in TBI-VEH, and 7% in TBI-DUO animals during the first post-injury week. A Kruskal–Wallis test followed by a Mann-Whitney *U*-test indicated no difference between the groups at any time-point (*p* > 0.05, Figure 4). A Wilcoxon signed-rank test indicated that sham-operated rats returned to their D0 weight (Sham-VEH 349 ± 7 g; Sham-DUO 345 ± 7 g) by D11 post-TBI (Sham-VEH 346 ± 5 g; Sham-DUO 337 ± 4 g, both *p* > 0.05 compared with D0). Rats with TBI began to gain weight on days 5–6 post-TBI, but did not reach their D0 weight (TBI-VEH 352 ± 5 g; TBI-DUO 360 ± 6 g) during the 14 d follow-up (TBI-VEH 338 ± 9 g; TBI-DUO 344 ± 7 g, both *p* < 0.05 compared with D0).

#### 2.2.2. Effect of Treatment on Behavior

##### Neuroscore

The Friedman test followed by Dunn’s post-hoc test indicated that during the 14 d follow-up, both the TBI-VEH and TBI-DUO groups exhibited impaired somatomotor performance on D2 and D6 post-injury as compared with baseline (both *p* < 0.01). Also, sham-operated rats were mildly impaired on D6 and D13 compared with baseline (both VEH and DUO groups *p* < 0.05).

Assessment of the neuroscore at each time-point separately revealed an injury effect on the neuroscore both in the injured VEH and DUO groups on D2 (TBI-VEH 11.4 ± 1.2 vs. Sham-VEH 25.0 ± 0.7, *p* < 0.01; TBI-DUO 10.8 ± 1.1 vs. Sham-DUO 24.4 ± 0.7, *p* < 0.001) and D6 (TBI-VEH 16.9 ± 1.0 vs. Sham-VEH 24.5 ± 0.5, *p* < 0.001; TBI-DUO 16.5 ± 1.1 vs. Sham-DUO 23 ± 1.1, *p* < 0.01). On D13 post-TBI, no injury effect was observed in the TBI-VEH group (TBI-VEH 19.0 ± 1.0 vs. Sham-VEH 23.2 ± 1.0, *p* > 0.05) whereas the TBI-DUO group remained mildly impaired (TBI-DUO 19.1 ± 1.3 vs. Sham-DUO 24.9 ± 0.3, *p* < 0.01).

**Figure 5:**
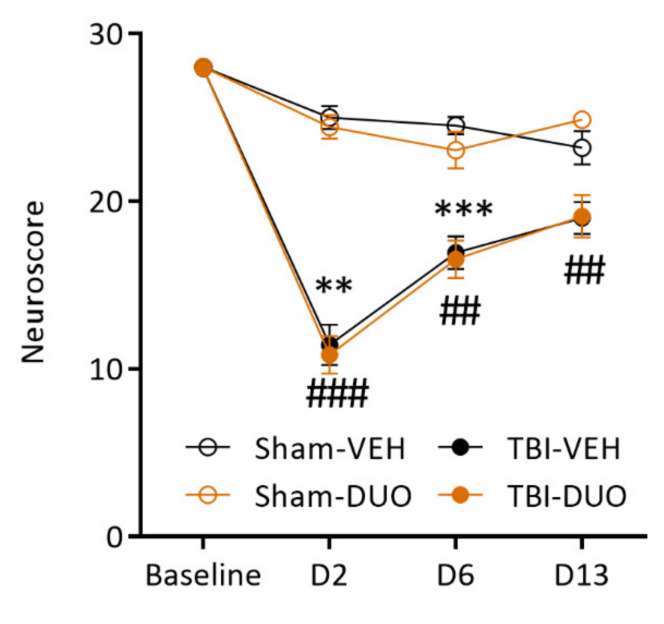
Composite neuroscore in rats treated with vehicle or SFN+NAC duotherapy. The TBI-VEH group showed impairment on day (D) 2 and D6 post-injury and the TBI-DUO group showed impairment on all testing days till D13 post-TBI. No difference was detected between Sham-VEH and Sham-DUO or TBI-VEH and TBI-DUO groups at any time-point (*p* > 0.05). Abbreviations: D, day; DUO, duotherapy treated with N-acetylcysteine and sulforaphane; NAC, N-acetylcysteine; SFN, sulforaphane; TBI, traumatic brain injury; VEH, vehicle. Statistical significances: ** *p* < 0.01, *** *p* < 0.001 (Sham-VEH vs. TBI-VEH, Mann–Whitney *U*-test); ## *p* < 0.01, ### *p* < 0.001 (Sham-DUO vs. TBI-DUO, Mann–Whitney *U*-test). Data are presented mean ± SEM.

The Friedman test followed by Dunn’s post-hoc test indicated no treatment effect on recovery of the neuroscore over the 14 d follow-up in the TBI groups (*p* > 0.05). Also, in the mildly impaired sham group, rats treated with duotherapy performed no better than the VEH-treated animals (*p* > 0.05, Figure 5).

Recovery index. Recovery between the follow-up days was compared between the TBI-VEH and TBI-DUO groups. A Mann–Whitney *U*-test indicated no treatment effect on recovery at D6-D2 (TBI-DUO 5.7 vs. TBI-VEH 4.2, *p* > 0.05), D13-D6 (TBI-DUO 2.6 vs. TBI-VEH 2.5, *p* > 0.05) or D13-D2 (TBI-DUO 8.3 vs. TBI-VEH 6.7, *p* > 0.05).

##### Beam Walking

Beam score. A paired sample t-test indicated that both the TBI-VEH and TBI-DUO groups were impaired in the beam walking test compared with their baseline values on D2 (both *p* < 0.01), D6 (both *p* < 0.05), and D13 (both *p* < 0.05) post-TBI. Sham-operation did not affect beam walking performance in the Sham-VEH or Sham-DUO groups (*p* > 0.05).

Repeated measures ANOVA followed by the Tukey post-hoc test indicated an injury effect on beam walking in the VEH and DUO groups (TBI-VEH vs. Sham-VEH, *p* < 0.05, TBI-DUO vs. Sham-DUO, p<0.05). An independent sample t-test revealed a difference on D2 (TBI-VEH 2.3 ± 0.7 vs. Sham-VEH 5.5 ± 0.1, *p* < 0.001; TBI-DUO 2.2 ± 0.6 vs. Sham-DUO 5.5 ± 0.2, *p* < 0.001), D6 (TBI-VEH 3.0 ± 0.8 vs. Sham-VEH 5.7 ± 0.1, *p* < 0.01; TBI-DUO 2.9 ± 0.6 vs. Sham-DUO 5.4 ± 0.3 vs, *p* < 0.01), and D13 (TBI-VEH 2.8 ± 0.7 vs. Sham-VEH 5.5 ± 0.1, *p* < 0.01; TBI-DUO 3.9 ± 0.6 vs. Sham-DUO 5.6 ± 0.1, *p* < 0.05) post-TBI. 

**Figure 6:**
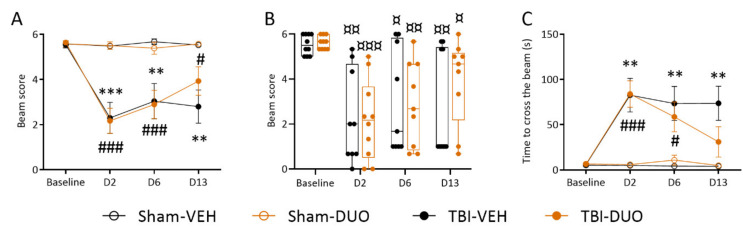
Beam walking in rats treated with vehicle or SFN+NAC duotherapy. (**A**) Both TBI-VEH and TBI-DUO groups were impaired in the beam walking test until day (D) 13 post-TBI. (**B**) A whisker-plot showing the distribution of scores in each animal throughout the 2-week follow-up. Interestingly, rats in the TBI-VEH group tended to cluster into the poorly-performing group (6 of 10 rats [60%] scoring 0-1) and rats in the TBI-DUO group into well-performing group (6 of 9 rats [67%] scoring 4-6), particularly at the end of the testing period. (**C**) The TBI-VEH group was impaired in beam crossing throughout the 2-week follow-up. The TBI-DUO group improved after D2, and did not differ from the Sham-DUO group on D13. Abbreviations: D, day; DUO, duotherapy treated with N-acetylcysteine and sulforaphane; NAC, N-acetylcysteine; SFN, sulforaphane; TBI, traumatic brain injury, VEH, vehicle; wk, week. Statistical significances: ** *p* < 0.01, *** *p* < 0.001 (Sham-VEH *vs.* TBI-VEH, independent sample *t*-test); # *p* < 0.05, ### *p* < 0.001 (Sham-DUO vs. TBI-DUO, independent sample *t*-test); ¤ *p* < 0.05, ¤¤ *p* < 0.01, ¤¤¤ *p* < 0.001 as compared to corresponding baseline (paired sample t-test). Data are presented as mean ± SEM or as whisker-plots with mean and minimum/maximum.

Repeated measures ANOVA followed by the Tukey post-hoc test indicated no treatment effect on recovery in the beam walking test throughout the 14 d follow-up between Sham-DUO and Sham-VEH or TBI-DUO and TBI-VEH groups (*p* > 0.05, Figure 6A). 

Interestingly, performance on the beam on D13 post-TBI divided animals into 2 groups (Figure 6B). In the well-performing group, rats walked the beam with no or less than 50% of foot slips (scores 4-6). In the poor-performing group, rats fell down or remained sitting on the beam (scores 0-1). In the TBI-VEH group, 40% (4/10) of the rats were well-performers (score 4-6) and 60% (6/10) were poor-performers (score 0–1). In the TBI-DUO group, percentages were 67% (6/9) and 22% (2/9), respectively (no treatment effect, chi square test *p* > 0.05). 

Recovery index. Recovery between the follow-up days was calculated for both TBI groups. There was no treatment effect on D6-D2 (TBI-DUO 0.63 vs. TBI-VEH 0.96, *p* > 0.05), D13-D6 (TBI-DUO 0.50 vs. TBI-VEH –0.04, *p* > 0.05), or D13-D2 recovery (TBI-DUO 1.52 vs. TBI-VEH 0.70, *p* > 0.05).

Time to cross the beam. A paired sample t-test indicated that rats in both the TBI-VEH and TBI-DUO groups passed the beam more slowly on D2 and D6 post-injury compared with baseline (both *p* < 0.01). The TBI-VEH group remained impaired even on D13 post-TBI (*p* < 0.05), whereas performance in the TBI-DUO group recovered to the baseline level (*p* > 0.05) (Figure 6C). 

Repeated measures ANOVA followed by the Tukey post-hoc test indicated an injury effect on beam walking in the VEH and DUO groups (TBI-VEH vs. Sham-VEH, *p* < 0.05; TBI-DUO vs. Sham-DUO, *p* < 0.05). An independent sample t-test revealed differences on D2 (TBI-VEH vs. Sham-VEH, *p* < 0.01; TBI-DUO vs. Sham-DUO, *p* < 0.001), D6 (TBI-VEH vs. Sham-VEH, *p* < 0.01; TBI-DUO vs. Sham-DUO, *p* < 0.05), and D13 for only the TBI-VEH group (TBI-VEH vs. Sham-VEH, *p* < 0.01; TBI-DUO vs. Sham-DUO, *p* > 0.05). 

Repeated measures ANOVA followed by the Tukey post-hoc test indicated no treatment effect on recovery in the beam walking test throughout the 14 d follow-up between the Sham-DUO vs. Sham-VEH or TBI-DUO vs. TBI-VEH groups (*p* > 0.05, Figure 6C).

#### 2.2.3. Effect of Treatment on Cortical Lesion Area

**Figure 7:**
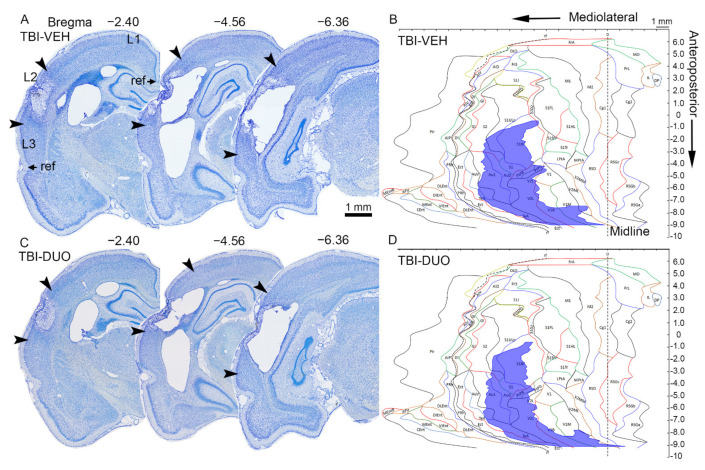
Location and area (mm^2^) of the cortical lesion. (**A**) Representative thionin-stained coronal sections showing the rostrocaudal extent of the cortical lesion in a rat in the TBI-VEH group on day (D) 14 post-TBI. Arrowheads indicate the mediolateral lesion extent at different bregma levels (bregma –2.40 to –6.36). L1 is the length of the cortical surface from the medial reference point (arrow, cortical contact with the corpus callosum) to the upper edge of the lesion, L2 is the mediolateral extent of the lesion, L3 is the distance from the lower edge of the lesion to the rhinal fissure (arrow). (**B**) Computer-generated unfolded cortical map showing the lesion location on the cortical mantle (violet) from the same animal. (**C**) Cortical lesion of a rat in the TBI-DUO group on D14 post-TBI. (**D**) Unfolded cortical map from the same animal. SFN+NAC duotherapy had no effect on the total lesion area or location (see Table 1). Scale bar equals 1 mm in all panels. Abbreviations: D, day; DUO, duotherapy treated with N-acetylcysteine and sulforaphane; NAC, N-acetylcysteine; SFN, sulforaphane; TBI, traumatic brain injury; VEH, vehicle. For abbreviations, see also Table 1.

Representative examples of unfolded cortical maps showing the location and extent of the cortical lesion in rats treated with vehicle or duotherapy are shown in Figure 7. The epicenter of the cortical lesion was mainly located in the auditory cortex, extending to the somatosensory and visual cortices (see Figure 7 and Table 1). Total lesion area did not differ between the TBI-DUO and TBI-VEH groups (TBI-DUO 24.87 ± 3.24 mm^2^; TBI-VEH 24.65 ± 3.21 mm^2^, *p* > 0.05). 

Also, the total lesion area or percentage of lesion in a given cytoarchitectonic cortical subfield did not differ between the TBI-DUO and TBI-VEH groups (all *p* > 0.05).

The cortical lesion area did not correlate with performance in the beam walking test (data not shown, *p* > 0.05). The greater the total lesion area, however, the poorer the performance in the neuroscore at D2 in TBI-VEH group (r = –0.69, *p* < 0.05), but not in the TBI-DUO group (r = –0.32, *p* > 0.05).

#### 2.2.4. Blood Cell, Plasma, and Brain Tissue Response Biomarkers

##### Blood Cells

Effect of systemically administered NAC+SFN on the expression of response biomarkers, that is, the Nrf2 encoding gene and its target genes (*Nfe2l2*, *Hmox1*, *Gclm*, *Nqo1*) in blood cells was assessed on D6 post-TBI, which was the last day of administration of both drugs.

Injury effect. In the TBI-VEH group, the expression of *Hmox1* increased to 188% compared with that in the Sham-VEH group (*p* < 0.05, Figure S1). No injury effect on the expression of *Gclm*, *Nqo1*, or *Nfe2l2* genes was detected (all *p* > 0.05, Figure S1). 

No injury effect on the expression of *Hmox1*, *Gclm*, *Nqo1*, or *Nfe2l2* genes was detected in the TBI-DUO group (all *p* > 0.05 as compared to the Sham-DUO group).

Treatment effect. In the craniectomized sham-operated experimental controls, the expression of *Gclm* in the Sham-DUO group was reduced to 14% of that in the Sham-VEH group on D6 post-operation (*p* < 0.05, Figure S1). No treatment effect on the expression of *Hmox1*, *Nqo1,* or *Nfe2l2* genes was detected (all *p* > 0.05). 

In injured animals, there was no difference in the expression of any of the analyzed genes between the TBI-DUO and TBI-VEH groups on D6 post-TBI (all *p* > 0.05, Figure S1). 

##### Plasma Markers

Plasma HMBG1 and cytokine levels were analyzed as response biomarkers for injury and treatment effects on neuroinflammation. 

Plasma quality. The mean ± SEM for A414 nm was 0.113 ± 0.003 (minimum/maximum 0.040 – 0.234).

HMGB1. The Kruskal–Wallis followed by the Mann–Whitney *U*-test indicated no difference in plasma HMGB1 levels between the animals randomized to different treatments at baseline (TBI-DUO 1.11 ± 0.50 ng/mL; TBI-VEH 1.36 ± 0.49 ng/mL, *p* > 0.05).

The Wilcoxon signed-rank test indicated no injury effect on plasma HMGB1 levels on D6 or D13 compared with baseline (*p* > 0.05, Figure S2). Also, no treatment effect was found on D6 (TBI-DUO 1.58 ± 0.35 ng/mL; TBI-VEH 1.53 ± 0.62 ng/mL *p* > 0.05) or D13 (TBI-DUO 2.78 ± 1.69 ng/mL; TBI-VEH 0.55 ± 0.21 ng/mL, *p* > 0.05) post-TBI.

Cytokines. Overall, the cytokine levels were at the lower end of the detection limit (Figure S3). A Kruskal–Wallis test indicated no TBI effect on D6 or D13 when the TBI-VEH group was compared with the Sham-VEH group or the TBI-DUO group was compared with the Sham-DUO group (*p* > 0.05). Interestingly, plasma cytokine levels in the Sham-VEH animals did not differ from baseline values on D6 or D13, indicating no long-lasting effect of anesthesia, craniectomy, repeated injections, or other procedures on cytokine levels. 

On D6 in the Sham-DUO group, a Mann–Whitney *U*-test indicated a treatment effect on plasma IL-6 and TNF levels compared with baseline values (IL6: D6 1515 ± 581 pg/mL vs. baseline 2766 ± 369 pg/mL, p< 0.05; TNF D6 1190 ± 554 pg/mL vs. baseline 1735 ± 276 pg/mL, *p* < 0.05). Comparable trends towards reduced levels were also seen for the other cytokines on D6 (*p* > 0.05) (Figure S3). On D13, that is 7 d after discontinuation of NAC, no treatment effect was detected. 

On D6, the Mann–Whitney *U*-test indicated a treatment effect also when plasma levels of IL-6 and TNF, as well as IL-1α, IL-2, IL-4, IL-5, IL-10, IL-12, IL-13, and INF-γ were compared between the Sham-DUO group and the Sham-VEH group (all, p<0.05).

No treatment effect on cytokine levels was detected in the TBI-DUO group on D6 or D13 (TBI-DUO vs. TBI-VEH, all *p* > 0.05).

##### Tissue Response Biomarkers

Cortical Nrf2 and platelet-derived growth factor receptor (PDGFR) β protein levels and cellular location were assessed as tissue response biomarkers for injury and treatment effects. 

**Figure 8:**
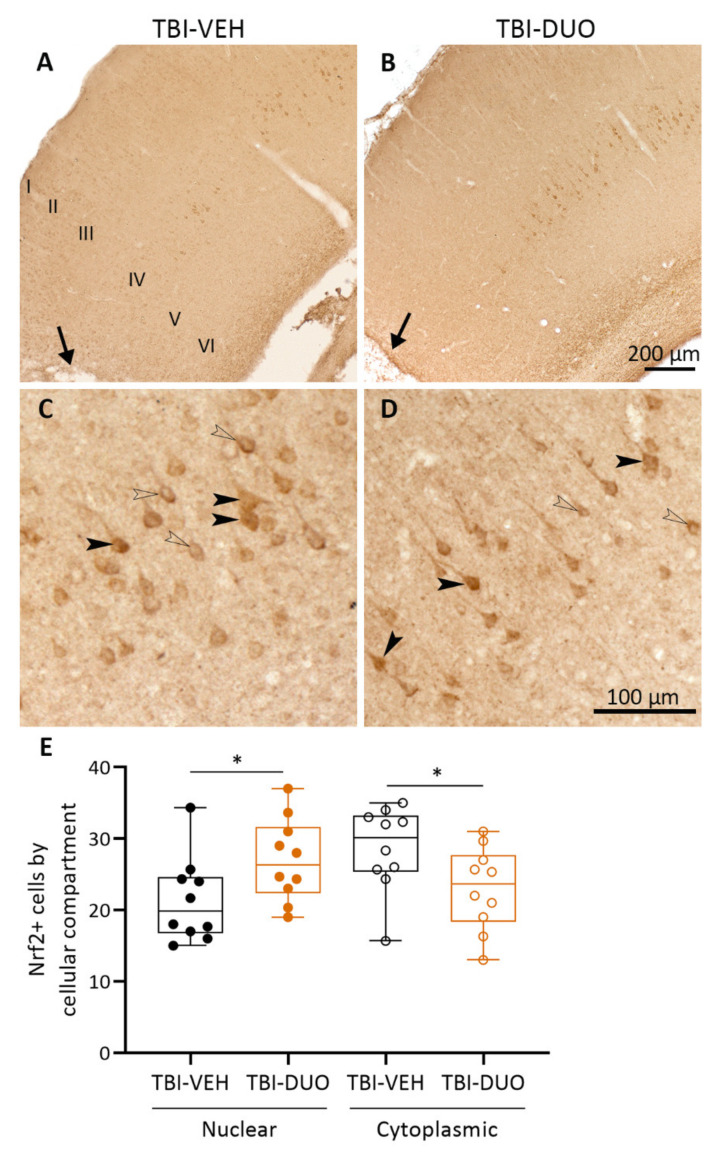
Nrf2+ immunostaining in the ipsilateral cortex. Low magnification photomicrographs showing Nrf2+ immunostaining mainly in cortical layer IV in (**A**) TBI-VEH and (**B**) TBI-DUO animals on day (D) 14 post-TBI. Note the lack of immunopositive neurons in a ~1-mm wide sector from the lesion core (lesion core is indicated with an arrow in panels A and B). Cortical layers are labeled with Roman numerals. High magnification photomicrographs showing nuclear (closed arrowhead) and cytoplasmic (open arrowhead) staining of Nrf2 in (**C**) TBI-VEH and (**D**) TBI-DUO animals. (**E**) Quantitative analysis indicated that the duotherapy increased nuclear localization and decreased cytoplasmic localization of Nrf2. Abbreviations: D, day; DUO, duotherapy treated with N-acetylcysteine and sulforaphane; Nrf2, nuclear factor erythroid 2-related factor-2; TBI, traumatic brain injury; VEH, vehicle. Statistical significances: * *p* < 0.05 Student’s *t*-test. Data are presented as whisker-plots with mean and minimum/maximum. Scale bar equals 200 µm in panels A–B and 100 µm in panels C–D.

Nrf2 immunostaining. Representative photomicrographs showing the distribution of Nrf2+ staining in the ipsilateral cortex dorsal to the lesion on D14 in TBI-VEH and TBI-DUO treated rats are shown in Figure 8A,B. Nrf2+ cells were mainly seen in the cortical layer IV. Both the neuronal cytosol and nucleus showed immunoreactivity (Figure 8C,D). Quantitative analysis indicated that the expression of Nrf2 was more prominent in the nucleus than in the cytoplasm in the TBI-DUO group compared with the TBI-VEH group (*p* < 0.05, Figure 8E).

**Figure 9:**
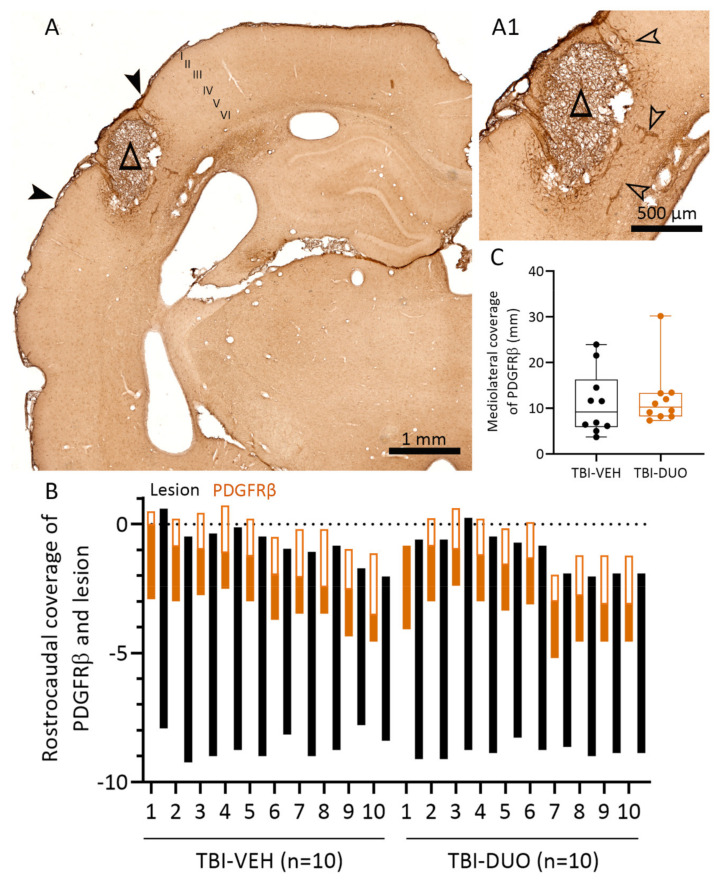
PDGFRβ+ immunostaining near the lesion core. (**A**) Low magnification photomicrograph showing PDGFRβ-immunolabeling on day (D) 14 post-TBI. Cortical layers are labeled with Roman numerals. (**A1**) High magnification photomicrograph showing prominent PDGFRβ immunoreactivity in blood vessel-related cells (open arrowheads) adjacent to the developing scar. (**B**) Rostrocaudal extent of the presence of PDGFRβ immunostaining (y-axis) in the anterior aspect of the lesion, where the staining was most prominent (10 TBI-VEH, 10 TBI-DUO, x-axis). “0” refers to bregma. Black bar indicates the rostrocaudal extent of the lesion. A bar with an orange outline shows the extent of the analysis of PDGFRβ-immunolabeling (filled orange bar: PDGFRβ positive labeling detected; unfilled orange bar: no labeling). (**C**) Quantification of mediolateral coverage (mm) of PDGFRβ-immunolabeling. The whisker-plot (mean, minimum/maximum) shows the total length of PDGFRβ-coverage in 10 sections (~2 mm; length between the closed arrowheads in panel A). No difference was detected between the TBI-VEH and TBI-DUO groups (*p* > 0.05, Mann–Whitney *U*-test). Abbreviations: D, day; DUO, duotherapy treated with N-acetylcysteine and sulforaphane; PDGFRβ, platelet-derived growth factor receptor β; TBI, traumatic brain injury; VEH, vehicle.

PDGFRβ immunostaining. A representative photomicrograph showing the expression pattern of PDGFRβ in the ipsilateral cortex on D14 post-TBI is shown in Figure 9A. We previously demonstrated PDGFRβ expression in parenchymal cells (astrocytes and NG2 cells), pericytes, and blood vessel-related cells in the injured brain [29]. PDGFRβ immunoreactivity was mainly seen in blood vessel-related cells in the vicinity of the developing scar in both TBI-VEH and TBI-DUO treated rats (Figure 9A,A1). No treatment effect was found in the rostrocaudal or mediolateral coverage of PDGFRβ between the TBI-VEH and TBI-DUO groups (all *p* > 0.05).

## 3. Discussion

Our objective was to assess the disease-modifying effect of antioxidant polytherapy on the outcome of TBI. We hypothesized that combined treatment with NAC and SFN would be neuroprotective, reduce inflammation, and mitigate post-TBI functional deficits. We found that NAC+SFN duotherapy robustly reduced nitrite levels and neuroinflammation, and increased neuronal viability in vitro. Subsequent in vivo validation indicated that duotherapy mitigated post-TBI impairment in the beam walking test. The effects on tissue and plasma biomarkers, however, were meager.

### 3.1. In Vitro Analysis Showed Comparable Efficacy of NAC+SFN Duotherapy and SFN Monotherapy

We first validated the in vitro effects of NAC and SFN monotherapies as well as NAC+SFN duotherapy on neurodegeneration and neuroinflammation, which are the hallmarks of secondary pathology following TBI [30]. Our in vitro study using neuronal and BV-2 microglial co-culture showed that NAC+SFN duotherapy decreased nitrite levels and neuroinflammation, and increased neuronal viability. The effects of duotherapy, however, did not exceed the effects of SFN monotherapy. Previous reports demonstrated that pre-treatment with SFN monotherapy provides neuroprotection against oxidative stress induced by hydrogen peroxide [31] or oxygen-glucose deprivation/reoxygenation [26,32] in vitro. Furthermore, SFN suppresses the mRNA and protein levels of proinflammatory mediators such as TNF, IL-6, and iNOS after LPS-induced neuroinflammation in vitro [21,33,34].

Interestingly, NAC treatment alone had no effect on neuronal viability or nitrite levels under neuroinflammatory conditions. This is contradictory to the findings of Chao and colleagues [35], who reported a protective effect of NAC against LPS-induced neurotoxicity in vitro. We found, however, that pretreatment with NAC resulted in decreased TNF levels after LPS-induced neuroinflammation, as was also reported by [36]. Taken together, our in vitro studies demonstrated favorable effects of both SFN and NAC on in vitro outcome measures. As the two drugs target somewhat different molecular pathways, however, we considered that administering SFN and NAC as a duotherapy would optimize the treatment effect.

### 3.2. In Vivo Analysis Demonstrated Mild Mitigation of Post-TBI Functional Impairment by NAC+SFN Duotherapy 

Recently, Pauletti et al., [13] demonstrated a disease-modifying effect of NAC+ SFN duotherapy on epileptogenesis and associated co-morbidities after SE. This finding encouraged us to apply the same treatment protocol to another epileptogenic etiology, TBI. In the present study, rats treated with NAC+SFN duotherapy showed mild improvement in the beam walking test, but no signs of increased recovery in the composite neuroscore test. The mild favorable behavioral outcome was not associated with a reduction in the cortical lesion area assessed on D14 post-TBI. Thus, the favorable in vitro effect was only partially validated in a preclinical in vivo model of severe TBI. Whether or not the use of a less severe TBI model, longer treatment period, or the selection of more sensitive outcome measures (e.g., using automated behavioral assessment instead of robust behavioral score-based assays) would result in the detection of beneficial treatment effects remains to be explored further.

Previous studies reported somewhat contradictory data on the efficacy of NAC or SFN monotherapies on TBI and stroke recovery in vivo. Beneficial effects of NAC monotherapy were reported on lesion size after lateral FPI [14,15], edema formation in a weight-drop model [16,37] and functional outcome in lateral FPI and weight-drop models [38,39]. Furthermore, NAC monotherapy decreased the infarct volume after stroke [40,41]. In a CCI model of TBI, however, NAC monotherapy did not improve cognition [42,43] or reduce tissue loss [44]. In another CCI study, NAC monotherapy did not alleviate edema formation or reduce lesion volume in a rat model [45]. Overall, our present in vitro data showing the effect on the neuroinflammatory component of the secondary post-TBI damage is consistent with the previous in vivo data in various injury models. 

Similar to findings using NAC, proof-of-concept SFN monotherapy studies have produced conflicting data. SFN is reported to decrease oxidative stress [27] and edema [46], protect BBB and tight-junctions [47], improve spatial memory or neurologic function [27,48], and reduce lesion volume [27] after CCI-induced TBI. A single dose of SFN also decreased infarct volume when assessed at 3 d after transient middle cerebral artery occlusion in rats [49]. Porritt and colleagues [50], however, reported that SFN was not neuroprotective when administered as a single dose or repeated dose over a 3 d period after photothrombosis-induced permanent cerebral ischemia; nor did it alleviate functional deficits.

Importantly, both treated and untreated groups showed similar post-impact apnea and righting reflex times, indicating that the treatment groups did not differ at baseline and thus the differences relate to the treatment effect. Also, we detected no adverse events of the drug treatment, such as excessive weight loss or diarrhea. The available data regarding the ability of NAC to cross the BBB are also contradictory. Some reports consider the BBB penetration to be low in normal brain [51]. Other studies suggest that due to the injury, NAC has access through the BBB [52]. In children with severe TBI a co-treatment with NAC and with its concentration increasing compound, probenecid, resulted in detectable levels of NAC in serum and delayed presence in CSF with no co-occurring adverse events [53]. As the authors state, the delay could relate to a poor BBB permeability of NAC. The brain NAC concentration might also depend on the dose administered. In a middle cerebral artery occlusion model of ischemic stroke, Khan and colleagues [41] showed that NAC did not provide protection at very low (50 mg/kg) or high (500 mg/kg) doses, but was highly protective when administered systemically at doses of 150 mg/kg and 250 mg/kg. Here we used systemic delivery of SFN and NAC into the injured brain during the first 2 post-injury weeks with doses that were anticipated to reach the brain levels ensuring target engagement. The favorable effects on functional recovery were milder than anticipated compared with previous studies in TBI and other injury models. Further studies are needed to assess whether lower efficacy of SFN+NAC duotherapy in the TBI model relates to differences in BBB penetration and target engagement rather than the severity and type of injury between models. 

### 3.3. Effect on Tissue Response Biomarkers 

We also analyzed two tissue biomarkers related to mechanisms of TBI-induced damage. TBI induces an increase in Nrf2 mRNA, Nrf2-related proteins, and the translocation of Nrf2 to the nucleus in rodents [27,54] and in humans [55]. Also, SFN can induce Nrf2 translocation to the nucleus [56], providing a tissue biomarker to monitor the treatment effect. 

Consistent with previous studies, we observed increased nuclear translocation of Nrf2 in the perilesional cortex of TBI animals treated with NAC+SFN duotherapy, suggesting that the treatment leads to increased transcription. Treatment after TBI, however, had no additional effect on the expression of Nrf2-encoding genes or its target genes measured from blood cells at D6 post-TBI. Others have reported acute changes in the Nrf2 target gene *Nqo1* in the TBI brain treated with SFN. Miller and colleagues [57] reported increased expression of *Nqo1* in the cortex up to 1 week after CCI. Furthermore, Zhao and colleagues [56] demonstrated a 1.3-fold increase in *Nqo1* expression in the striatum within 3 h after intracerebral hemorrhage injury and SFN injection, whereas Hong and colleagues [27] showed a one-fold increase of the same gene in the cortex after CCI and an additional 0.2-fold increase within 24 h after SFN treatment. Assessment of the coverage of PDGFRβ positivity in the perilesional area, a marker of the wound healing capacity after injury [29,58], showed no treatment effect. 

Thus, biomarker analysis indicated a mild effect of the duotherapy on tissue response biomarkers, supporting the idea that the systemically administered small molecules reached the perilesional area as expected.

### 3.4. Effect on Plasma Response Biomarkers

We failed to detect an injury effect on plasma HMGB1 levels when assessed on D6 and D13 post-TBI. This may be due to early release of HMGB1 following TBI. Consequently, timing of the analysis could have been too late. This is supported by clinical data showing that HMGB1 levels in the plasma [59,60] become elevated within hours after TBI in adults, as well as in the CSF [61] and serum [62] in children. Because no injury effect was detected in this study, our ability to discover any treatment effect was also compromised.

Previous studies demonstrated acutely (within hours to days) and more chronically (by 3 to 6 months) increased cytokine levels in the brain and plasma or serum after TBI [16,17,63,64,65]. As our in vitro data revealed that NAC+SFN duotherapy decreased the inflammation-induced increase in TNF levels, we tested the hypothesis that NAC+SFN duotherapy would also reduce markers of neuroinflammation in vivo. We did not detect major TBI- or treatment-related changes in plasma response biomarkers, but the expression pattern of the same biomarkers was not measured from brain tissue in this study. Interestingly, Kamm and colleagues [64] reported that IL-1β and IL-10 were increased in the brain post-TBI, but at the same time were absent in plasma. This may be due to a different time course of the peripheral and CNS cytokine load following brain injury.

In general, the profile of plasma cytokine levels on D6 post-operation was similar between treatment groups, being close to the lower limits of detection. Interestingly, however, NAC+SFN duotherapy decreased plasma IL-6 and TNF levels on D6 in craniectomized sham-operated rats. Even though sham-operation, including anesthesia and craniectomy, is commonly used as an experimental control, it reported to induce, for example, subcraniectomy astrogliosis, inflammation, and blood extravasation into the brain parenchyma [66,67,68], mimicking a mild TBI. Our data suggest that NAC+SFN duotherapy showed neuroprotection in craniectomized animals. Due to the robustness of the severe TBI, no effect was observed in the TBI-DUO group. Interestingly, both NAC and SFN target nuclear factor κB (NFκB) activation, and consequently, proinflammatory cytokine production such as IL-6 and TNF from different aspects. NAC mediates the activation of NFκB by suppressing both IκB kinases (IKK) α and IKKβ while SFN operates through Nrf2 by preventing the direct binding of NFκB and DNA [69,70,71]. Because we found decreased levels of TNF only on D6 during NAC+SFN duotherapy and not on D13 during SFN monotherapy, we suggest that these two mechanisms are both needed to effectively suppress TNF levels after injury in vivo.

### 3.5. Caveats

Even with the strong in vitro efficacy of a drug, the in vivo validation process may not be sensitive enough to demonstrate favorable in vivo effects [72]. As our in vitro study indicated, not only monotherapy with SFN but also monotherapy with NAC had anti-inflammatory potential. Therefore, we proceeded with duotherapy with NAC and SFN rather than SFN monotherapy for an in vivo proof-of-concept study. The overall in vivo disease-modifying effect, however, was milder than expected based on the in vitro data. The net in vivo effect can be the sum of many factors such as the timing of treatment, type and severity of the injury, or simply the complexity of the molecular pathology of the injured brain. Interestingly, though, we detected a similar treatment-related decrease in the expression of TNF in in vitro and in vivo models.

Single housing can increase stress and affect cytokine levels in rats [73,74]. To keep the living conditions stable over time, all sampling and testing were performed while animals were single housed. Moreover, living conditions were similar for all treatment groups.

Brain insults such as TBI or SE can lead to structural epilepsy, resulting in spontaneous recurring seizures [75]. Our overarching objective was to test the hypothesis that a favorable antiepileptogenic treatment in one model of structural epilepsy could be applied to improve the outcome in another epileptogenic etiology. TBI and SE share many mechanistic features such as neuroinflammation, oxidative stress, and neuronal loss, which can be targeted with antioxidant therapy [76]. SE models of epileptogenesis have an advantage over TBI models because epileptogenesis is faster, more animals develop epilepsy, and seizure frequency is higher [77]. Therefore, SE models are often used as the first proof-of-concept in vivo testing platform for new antiepileptogenesis candidates.

Pauletti and colleagues [13] reported that duotherapy with NAC and SFN rescued mitochondrial dysfunction, decreased oxidative stress, and reduced pathologically generated HMGB1 in the brain and blood by D4 after induction of SE. Thus, the same antioxidant drugs and experimental setting was used in the present study, but we were unable to confirm HMGB1-related changes in our model at a slightly delayed time-point of D6 post-TBI. Furthermore, the antioxidant duotherapy starting early after SE provided neuroprotection by D4, rescued cognitive deficits in a working memory test, and resulted in long-term seizure control within 5.5 months post-SE [13]. In the present study, we detected mild behavioral improvement by D13 after treatment that was not associated with neuroprotection by D14 post-TBI. Whether or not the reduction in chronic spontaneous seizure activity after electrical SE reported by Pauletti and colleagues [13] also occurs after TBI would be an interesting topic for future studies.

## 4. Materials and Methods

### 4.1. In Vitro Assessment of Neuroprotective, Antioxidant, and Anti-Inflammatory Effects of SFN, NAC, and Their Combinations

#### 4.1.1. Preparation of Mouse Primary Cortical Neurons and BV-2 Cell Co-Culture 

Cortical neuronal culture. Brains were dissected from JAXC57BL/6J mice on embryonic day 18 (E18) and the cerebral cortex was carefully stripped of the meninges. Cortical tissue was digested with 0.125% trypsin (#15090046, Gibco, Thermo Fisher Scientific, Waltham, MA, USA) in Dulbecco’s Modified Eagle’s Medium (DMEM, #BE12-614F Lonza, Bale, Switzerland) for 20 min at 37 °C. The trypsin reaction was stopped using plating medium containing 10% fetal bovine serum (FBS, #10270-106, Gibco, Thermo Fisher Scientific), 100 U/mL penicillin, and 100 μg/mL streptomycin (#DE17-602E Lonza) in DMEM. The digested cortices were centrifuged at 1600× *g* for 5 min at room temperature (RT) and the pellet was resuspended in plating medium. The cell suspension was filtered using a 40 µM cell strainer (#542040, Greiner Bio-One, Kremsmuenster, Austria). The filtrate was centrifuged at 1200× *g* for 5 min at RT and the remaining pellet was resuspended in neuronal culture medium containing Neurobasal™ feeding medium (#12348017, Gibco, Thermo Fisher Scientific) supplemented with 2% B27 (#17504044, Gibco, Thermo Fisher Scientific), 2 mM L-glutamine (#17-605E, Lonza), 100 U/mL penicillin, and 100 μg/mL streptomycin. Supplemented Neurobasal™ feeding medium was added to obtain a single cell suspension. Cortical neurons were counted using a hemocytometer and Neurobasal™ feeding medium was added to reach a final density of 40 × 10^4^ cells/mL. Then, 20 × 10^4^ cells in feeding medium were plated on 1× poly-D-lysine hydrobromide (#P6407, Sigma Aldrich, St Louis, MO, USA) -coated 48-well cell culture plates and cultured at 37 °C in a 5% CO_2_ humidified atmosphere. 

Co-cultures. Cortical neurons were co-cultured with BV-2-microglia cells after culturing the neurons for 5 d. Briefly, one confluent 15 cm plate of BV2-microglia cells cultured in RPMI™-1640 medium (#R0883, Sigma Aldrich) supplemented with 1.1% 100 U/mL penicillin, 100 μg/mL streptomycin, 1.1% L-glutamine, and 9% FBS was used. To eliminate dead BV2 cells, RPMI medium was removed, and cells were washed once with phosphate buffered saline (PBS). Then, BV2-microglia cells were gently scraped into fresh RPMI medium to create a cell suspension. The suspension was centrifuged at 800× *g* for 3 min at RT, and the remaining pellet was resuspended in the neuronal culture medium. Neuronal culture medium was then added to obtain a single cell suspension. BV2-microglia were counted using a hemocytometer to obtain a density of 20 × 10^4^ cells/mL. Next, neuronal culture medium was removed from each well, and BV2 microglia cells were seeded at a density of 40 × 10^3^ cells/mL in neuronal culture medium on top of the neurons to obtain a cortical neuron-BV2 microglia co-culture [78,79,80]. The final ratio of BV2-microglia to cortical neurons was 1:5.

#### 4.1.2. In Vitro Assessment of the Treatment Effects

Test drugs. At 1 h after adding the BV2-microglia cells, co-cultures were treated using monotherapy with either SFN or NAC at different concentrations or SFN+NAC duotherapy. SFN (#S8044, LKT Laboratories, St. Paul, MN, USA) was dissolved in 1% dimethyl sulfoxide (DMSO) in supplemented Neurobasal™ feeding medium at concentrations of 0.1 µM, 1 µM, 5 µM, and 10 µM [26,32,81]. NAC (A7250, Sigma Aldrich, St. Louis, MO, USA) was dissolved in H_2_O (pH was adjusted with 2 M or 10 M NaOH to 7.4) at concentrations of 1 µM, 10 µM, 100 µM, and 300 µM [35,82]. The SFN+NAC ratios in duotherapy were 1:10 [83], 1:30, and 1:100 [13].

Assay Controls. Anti-inflammatory cytokine IL-10 (50 µg/mL, #500-M128, Peprotech, Rocky Hill, USA; final concentration of 50 ng/mL) and neuroprotective iNOS inhibitor (1400 W hydrochloride, 2 mM, #1415, Tocris, Bristol, UK; final concentration of 20 µM) were used as positive controls [78]. 

Induction of neuroinflammation. After a 1 h drug treatment, neuroinflammation was induced by 200 ng/mL of LPS (#L5543, Sigma Aldrich) and 20 ng/mL INFγ (#i4777, Sigma Aldrich). At 48 h after the induction of neuroinflammation, cell culture supernatants were collected and stored at −20°C until further analysis of nitrite and TNF levels. As negative controls, we used eight control wells, four containing cortical neurons only and four containing both cortical neurons and BV-2-microglia cells without exposure to LPS/INFγ-induced neuroinflammation.

#### 4.1.3. In Vitro Outcome Measures

##### Neuronal Viability Assay

Co-culture cells were fixed with 4% paraformaldehyde (PFA, #28908, Thermo Fisher Scientific) in PBS for 20 min, and then washed twice with PBS. Fixed cells were stored in PBS, and plates were covered with parafilm and kept at 4 °C. The viability of the cortical neurons was assessed by microtubule-associated-protein 2 (MAP2) immunostaining, as described by Brooke et al., [28]. Briefly, cells were incubated in 0.3% H_2_O_2_ in methanol for 10 min at RT for permeabilization and to block endogenous peroxidase activity. Non-specific staining was blocked by incubating the cells in 1% bovine serum albumin (#A9647, Sigma Aldrich) and 10% normal horse serum (#S-2000, Vector Laboratories, Burlingame, CA, USA) in PBS for 20 min. This was followed by overnight incubation in primary mouse anti-MAP2 antibody (1:2000, M9942, Sigma Aldrich) in blocking serum at 4 °C. Biotinylated secondary horse anti-mouse antibody (1:2000, BA-2000, Vector Laboratories) in blocking serum was then added, and the mixture was incubated at RT for 1 h. For visualization of the reaction product, the wells were incubated for 1 h at RT with ExtrAvidin-HRP (1:2000, E2886, Sigma Aldrich) dissolved in blocking serum. The cells were washed three times for 10 min between incubations. TMB peroxidase (3,3’,5,5’-tetramethylbenzidine, #SK-4400, Vector Laboratories) was prepared in a darkroom or a room with red light following the manufacturer’s instructions, and added to the cells. After a 10-min incubation in TMB substrate, the substrate solution was transferred to a microtiter plate and absorbance was measured using a microtiter plate reader (Infinite M200, Tecan, (Grödig, Austria) at 650 nm and the Magellan program. The wells processed without primary antibody were used as background controls. The experiment was repeated twice, and data from both experiments were combined (two independent experiments, each with three replicates). The following equation was used for normalizing the results:

$Percentage\;of\;neuronal\;\;viability=\frac{absorbance\;\left(drug\;treated\;wells-LPS/INF\gamma +wells\right)}{absorbance\left(1400W\;treated\;wells-LPS/INF\gamma +wells\right)\;}$ *x 100%*

##### Nitrite Assay

To measure the amount of nitric oxide secreted into the cell culture medium, a nitrite assay was performed using a Griess reagent kit (#G-7921, Thermo Fisher Scientific, Molecular Probes). The kit detects nitrite that is formed by the spontaneous oxidation of nitric oxide present in the cell culture medium. To carry out the nitrite assay, standards were prepared from a stock solution following the manufacturer’s instructions. Samples were diluted in deionized water at a ratio of 1:4. Samples and standards were incubated in Griess reagent for 30 min at RT. Absorbance was measured at a wavelength of 548 nm using a microplate reader (Infinite M200, Tecan) and the Magellan program. The experiment was repeated twice and data from both experiment batches were combined (two independent experiments, each with four replicates). The following equation was used for normalizing the results:

$Percentage\;of\;nitrite=\frac{absorbance\;\left(drug\;treated\;wells-1400W\;treated\;wells\right)}{absorbance\left(LPS/INF\gamma +wells-1400W\;treated\;wells\right)}$ *x 100%*

##### TNF Enzyme-Linked Immunosorbent Assay (ELISA) from Cell Culture Medium

The concentration of TNF in the cell culture medium was detected using a mouse TNF enzyme-linked immunosorbent assay (ELISA) kit (#88-7324-22, Invitrogen, Thermo Fisher Scientific) according to the recommendations and dilutions given by the manufacturer. Absorbance was measured at a wavelength of 450 nm using the Infinite M200 Tecan plate reader and the Magellan program. The ELISA was repeated twice and data from both experiment batches were combined (two independent experiments, each with four replicates). The following equation was used for normalizing the results:

$Percentage\;of\;TNF=\frac{absorbance\;\left(drug\;treated\;wells-IL10\;treated\;wells\right)}{absorbance\left(LPS/INF\gamma +wells-IL10\;treated\;wells\right)}\;$ *x 100%*

### 4.2. In Vivo Assessment of Treatment Effects

#### 4.2.1. Lateral Fluid-Percussion Induced TBI

Animals. Male Sprague–Dawley rats were used (350 ± 30 g; Envigo, Horst, Netherlands, 12 ± 1 weeks old at the time of injury). Animals were single-housed (cage size 28.5 cm × 48.5 cm × 20 cm) in a controlled environment (temperature 22 ± 2°C, humidity 55% ± 15%, light-dark cycle from 07.00–19.00) with free access to food (Teklad 2016S) and water.

All animal procedures were approved by the Animal Ethics Committee of the Provincial Government of Southern Finland and performed in accordance with the guidelines of the European Community Council Directives 2010/63/EU.

Induction of lateral FPI. TBI was induced by lateral FPI according to McIntosh et al., [84]. Animals were anesthetized with isoflurane (5% induction, 2.2–2.7% maintenance, room air 500 ml/min, Kent Scientific SomnoSuite®), and fixed in a stereotaxic frame. Lidocaine (10 mg/ml, volume 0.1 ml, Orion Oyj) was injected subcutaneously in the region of planned craniectomy. A 5 mm craniectomy was created over the left cortex with a hand-held trephine midway between lambda and bregma, and midway between the sagittal suture and temporal ridge (craniectomy center: AP −4.5 mm; ML 2.5 mm; rat brain atlas [85]). The intactness of the dura was checked, and a plastic female Luer-lock connector made from 18G needle hub was placed into the craniectomy with its edges sealed with 3M tissue glue (3M Vetbond, 3M Deutschland GmbH, Germany). The connector was stabilized to the skull with a screw (1 mm, #BN82213, Bossard) placed rostral to bregma and surrounded by dental cement (Selectaplus, DeguDent GmbH, Germany). The rat was then removed from the stereotaxic frame and immediately connected to the FPI device (AmScien Instruments, model 302; Richmond, VA, USA) equipped with a straight tip. After the rat responded to a toe pinch, a pressure pulse (2.5 ± 0.2 atm) monitored with a PC-based automatic pressure measurement unit was delivered. Post-impact apnea time (s) was measured. The rat was moved onto a heating pad (+38 ^o^C) and placed on its right side, and the tongue was pulled to one side to facilitate breathing. Time to righting (min) was measured. The rat was re-anesthetized with 5% isoflurane (maintenance 2.2–2.7%), the injury cap was removed, and the incision was sutured. Buprenorphine (0.3 mg/ml, volume 0.15 ml, Orion Oyj) and 10 ml of 0.9% NaCl were administered subcutaneously (s.c.). Sham-operated controls underwent the surgery without the pressure pulse.

Post-operative monitoring. Weight was recorded on the day (D) of the injury (D0), and then daily throughout the 2-week follow-up (Scale 1, #PT1500; Scale 2, #BP3100; SARTORIUM Vendor). After the operation, powdered pellets of Teklad 2016S were provided as supplementary food for 1–3 days or as needed. The supplementary volume correction was delivered using 0.9% NaCl (20 ml/24 h, s.c.) for 1–3 days or longer if needed. Acute (<48 h) and follow-up (>48 h) mortality, occurrence of post-impact seizure-like behavior, and dural breaks were recorded. 

#### 4.2.2. Treatment Groups 

The dose and dosing of SFN and NAC was based on the study of Pauletti et al., [13] as our objective was to assess whether the treatment paradigm shown to be neuroprotective and disease-modifying in the SE model would have comparable effects after TBI. In a power analysis we anticipated a 20% difference between the vehicle and SFN+NAC groups with 10% standard deviation (power 0.8, *p* < 0.05). Consequently, a total of 40 rats were randomized into the sham-operation or TBI by lateral FPI groups (Figure 1B). The 34 surviving rats (14 sham-operated, 20 TBI) were randomized into 4 treatment groups. 

Sham-VEH (sham-operated experimental controls with vehicle treatment, n = 7): Starting at 1 h after sham-operation (D0), rats were treated with sterile H_2_O (intraperitoneally, i.p.) twice a day (6 h apart) on D0–D6. On D0–D6, at 1 h after the first H_2_O injection, rats were also administered 0.1% DMSO in buffered saline (i.p.). On D7-D13, rats were treated with a single dose of 0.1% DMSO in buffered saline (i.p.).

Sham-DUO (sham-operated experimental controls with NAC+SFN duotherapy, n = 7): Starting at 1 h after sham-operation (D0), rats were treated with NAC (500 mg/kg, dissolved in sterile H_2_O, pH 7.4, i.p.) twice a day (6 h apart) on D0–D6. On D0–D6, at 1 h after the first NAC injection, rats were administered SFN (5 mg/kg, dissolved in 0.1% DMSO in buffered saline, pH 7.4, i.p.). On D7–D13, rats were treated with a single dose of SFN only (i.p.).

TBI-VEH (TBI animals with vehicle treatment, n = 10): Starting at 1 h after TBI (D0), rats were treated with sterile H_2_O (i.p.) twice a day (6 h apart) on D0–D6. On D0–D6, at 1 h after the first H_2_O injection, rats were administered 0.1% DMSO in buffered saline (i.p.). On D7–D13, rats were treated with a single dose of 0.1% DMSO in buffered saline (i.p.).

TBI-DUO (TBI animals with NAC+SFN duotherapy, n = 10): Starting at 1 h after TBI (D0), rats were treated with NAC (500 mg/kg, dissolved in sterile H_2_O, pH 7.4, i.p.) twice a day (6 h apart) on D0–D6. On D0–D6, at 1 h after the first NAC injection, rats were administered SFN (5 mg/kg, dissolved in 0.1% DMSO in buffered saline, pH 7.4, i.p.). On D7-D13, rats were treated with a single dose of SFN only (i.p.).

Drugs were prepared fresh daily close to the time of administration.

#### 4.2.3. Behavioral Analysis

Composite neuromotor score (neuroscore). Testing was performed at baseline, and then on D2, D6, and D13 after sham-operation or TBI. Animals were given a score from 0 (severely impaired) to 4 (normal) for each of the following seven indices: left and right (two indices) forelimb flexion during tail suspension (contra flexion), left and right (two indices) hind limb flexion when the forelimbs remained on the hard surfaces and the hind limbs were lifted up and back by the tail (hind limb flexion), ability to resist a lateral pulsion towards the left and right (two indices), and angle board (one index) as described by Nissinen et al., [86]. A composite neuroscore (0–28) was generated by combining the scores from each of the seven tests.

Recovery index for neuroscore. Recovery between follow-up days was calculated separately for the TBI-VEH and TBI-DUO groups by subtracting the neuroscore scores of D2 (48 h post-TBI) from D6, D6 from D13, and D2 from D13.

Beam walking. To evaluate complex motor movement and coordination, beam walking was evaluated according to Ohlsson and Johansson [87] at baseline, and then on D2 (48 h post-operation), D6, and D13 after TBI or sham-operation. Testing was performed after assessment of the neuroscore on the same day. Briefly, the rat was first habituated to the beam (1390 mm long and 21 mm wide wooden bar placed 430 mm above the floor) and the black box (250 × 200 mm), which was located at the right end of the beam. On the actual testing day, the rat was allowed to walk the beam 3 times and remain in the black box for 1 min between each run. Behavior on the beam was scored from 0 (falls down from the beam) to 6 (walks the beam without slipping), and the mean of the 3 runs was calculated. The time required for the rat to walk the beam was measured and mean of the 3 runs was calculated (each run took maximum 120 s). In cases in which the rat remained sitting on the beam (score 1) or fell down immediately (score 0) before crossing the beam completely (score 2), a total time of 120 s time was given.

Recovery index for beam walking. Recovery between the follow-up days was calculated separately for the TBI-VEH and TBI-DUO groups by subtracting the beam scores of D2 (48 h post-operation) from D6, D6 from D13, and D2 from D13.

#### 4.2.4. Blood and Plasma Biomarker Analysis

##### Blood Sampling

Sampling was performed at baseline, and then on D6 (144 h after operation) and D13 prior to drug administration according to van Vliet et al., [88]. Blood was sampled from the lateral tail vein using a 23G butterfly needle (#367284, BD Vacutainer) under brief isoflurane anesthesia (5% induction, 1–2% maintenance, air 500 mL/min). A total of 1 ml of blood was collected into two K2 ethylenediaminetetraacetic acid (EDTA)-coated tubes (500 µL blood per tube; #365975, BD Microtainer, BD Biosciences, Franklin Lakes, NJ, USA), mixed 10 times, and kept on ice until centrifuged. To separate plasma, samples were centrifuged at 4 °C 1300× *g* for 10 min. Samples were kept on ice and plasma was aliquoted into five 50 µl aliquots into Protein LoBind tubes (#022431064, Eppendorf), frozen on dry ice, and stored at –70 °C for further analysis. The remaining blood cells (leukocytes, erythrocytes cells, and platelets) were frozen on dry ice and stored at –70 °C for further analysis.

Plasma quality was assessed for each sample by measuring hemoglobin absorbance by spectrophotometry at 414 nm (NanoDrop® ND1000 Spectrophotometer, ND-1000 v3.8.1) and using a cut-off value of 0.25.

##### RNA Extraction and Reverse Transcription Quantitative Polymerase Chain Reaction (RT-qPCR)

To assess response biomarker gene expression in blood cells, expression of nuclear factor erythroid 2-like 2 (*Nfe2l2*, gene coding Nrf2), heme oxygenase 1 (*Hmox1*), NAD(P)H quinone oxidoreductase 1 (*Nqo1*), and glutamate-cysteine ligase modifier subunit (*Gclm*) was measured from tail vein blood cells collected on D6 (n = 7 per experimental group, in total 28 samples).

RNA extraction. Total RNA was extracted from blood cells using NucleoZol reagent (#740404.200, Macherney-Nagel, Düren, Germany) following the manufacturer’s instructions. After extraction, the RNA concentration was quantified using a NanoDrop UV-VIS spectrophotometer. To ensure a sufficient RNA quality, an Agilent Bioanalyzer RNA 6000 Nano Kit and 2100 Bioanalyzer (Agilent Technologies, Palo Alto, CA, USA) was used to measure RNA Integrity Number (RIN). The RIN value was between 6.5 to 8, which is consistent with previous data from blood cell samples [89], indicating good RNA quality.

RT-qPCR. cDNA was synthetized from 1 µg of RNA using the Bio-Rad iScript™ Advanced cDNA Synthesis Kit (#1725038, Bio-Rad, Hercules, CA, USA). RT-qPCR was carried out to amplify 10 µg of cDNA using Bio-Rad SsoAdvanced™ Universal SYBR® Green Supermix (#172-5272). Nrf2-encoding gene *Nfe2l2,* and its target genes *Hmox1*, *Nqo1*, and *Gclm* (#10041595, Bio-Rad) were amplified simultaneously with control rat glyceraldehyde 3-phosphate dehydrogenase (*Gapdh*). The RT-qPCR conditions were as follows (Roche LightCycle® 96): one cycle of preincubation (95 °C for 120 s), 40 cycles of two-step amplification (95 °C for 5 s and 60 °C for 30 s), one cycle of melting (95 °C for 5 s, 65 °C for 5 s, 95 °C for 1 s), and 1 cycle of cooling (37 °C for 30 s). The values were normalized relative to *Gapdh* (fold-change) and the relative fold-change of the analyzed genes was calculated using the 2 ^−ΔCT^ method [90].

##### Multiplex Analysis of Plasma Cytokine Levels

Expression of 12 rat cytokines (IL-1α, IL-1β, IL-2, IL-4, IL-5, IL-6, IL-10, IL-12(p70), IL-13, granulocyte-macrophage colony-stimulating factor [GM-CSF], TNF, and INF-γ) was evaluated from rat plasma samples obtained at baseline, and on D6 and D13 using a commercially available kit (Bio-Plex Pro™ rat cytokine Th1/Th2 assay #171k1002m, Bio-Rad Laboratories) according to the manufacturer’s instructions. Plates were analyzed using Bio-Plex 200 System with Bio-Plex 4.1.1 software (Bio-Rad).

##### ELISA for Plasma High-Mobility Group Box 1 (HMGB1) Levels

Total HMGB1 was measured from plasma samples obtained at baseline, D6 and D13 (TBI-VEH n = 6, TBI-DUO n = 7) using a commercially available ELISA kit II (Shino-test Corp, Sagamihara, Japan) according to the manufacturer’s instructions.

#### 4.2.5. Processing of Brain for Histology

##### Perfusion

On D14, rats were deeply anesthetized with an intraperitoneal injection of pentobarbital (60 mg/kg) and perfused transcardially via the aorta with 0.9% NaCl (6 min, 30 ml/min) followed by 4% PFA in 0.1 M phosphate buffer (PB), pH 7.4 (30 min, 30 ml/min). The brain was removed from the skull, fixed in 4% PFA for 4 h, cryoprotected in 20% glycerol in 0.02 M potassium phosphate-buffered saline (KPBS, pH 7.4) for 24 h, frozen on dry ice, and stored at −70 °C for further processing.

Frozen coronal sections of the brain were cut (30 µm thick, one-in-five series) using a sliding microtome. The first series of sections was stored in 10% formalin at RT and used for thionin staining. Other series of sections were collected into tissue collection solution (30% ethylene glycol, 25% glycerol in 0.05 M PB) and stored at −20 °C until processed.

##### Nissl Staining

The first series of sections was stained with thionin, cleared in xylene, and cover-slipped using Depex® (BDH Chemical, Poole, UK) as a mounting medium.

##### Cortical Unfolded Maps

To assess the severity and cytoarchitectonic distribution of the lesion after TBI, all thionin-stained sections were digitized (40×, Hamamatsu Photonics, NanoZoomer-XR, NDP.scan 3.2) and unfolded cortical maps were prepared to quantify the treatment effect on total cortical lesion area and the damage to different cytoarchitectonic cortical areas (see details from [91], in-house software at [92]).

##### Immunohistochemistry

The effect of antioxidant duotherapy on 2 tissue biomarkers, Nrf2 and PDGFRβ, was assessed. Previous studies demonstrated that the transcription of Nrf2 is activated by SFN [21,22]. PDGFRβ is involved in tissue healing processes after injury and is upregulated near the lesion site [29,58,93]. The expression of each marker was assessed on D14 in a 1-in-5 series of coronal sections containing the cortical lesion. The following antibodies were used: primary antibody polyclonal rabbit anti-human Nrf2 (1:1000, ab31163, Abcam, Cambridge, UK), primary antibody monoclonal rabbit anti-human PDGFRβ (1:2000, ab32570, Abcam), and biotinylated secondary antibody goat anti-rabbit IgG (1:300, BA1000, Vector Laboratories). The free-floating sections were rinsed three times in 0.02 M KPBS (10 min each). Endogenous peroxidase was removed by incubating sections for 15 min in 1% H_2_O_2_ in 0.02 M KPBS. Then, sections were washed (six times, 5 min each), and non-specific binding was blocked with a solution containing 10% normal goat serum (NGS) and 0.5% Triton-X-100 in 0.02 M KPBS for 2 h in RT. The sections were then incubated in a primary antibody solution in 1% NGS and 0.5% Triton X-100 in 0.02 M KPBS for 2–3 nights at 4 °C. After three washes (10 min each) in 2% NGS in 0.02 M KPBS, the sections were incubated in a secondary antibody solution in 1% NGS and 0.5% Triton X-100 in 0.02 M KPBS for 2 h in RT. Next, the sections were washed (three times, 10 min each) and incubated with avidin-biotin complex for 2 h at RT. Finally, after washing, the staining was visualized by incubating the sections in 0.1% 3′,3′-diaminobenzidine (Pierce Chemicals, Rockford, IL, USA) solution containing 0.04% H_2_O_2_ in 0.02 M KPBS. The sections were washed once with 0.02 M KPBS and twice in 0.1 M PB, mounted on gelatin-coated slides, and covered using DePex®.

Quantification of Nrf2+ immunostaining. Three consecutive sections covering the lesion epicenter (AP –2.76 to –3.00 mm from bregma) were analyzed from each rat. Dorsal to the cortical lesion core, a total of 50 Nrf2+ cells were included into the visual analysis under a Leica DMRB microscope. As an outcome measure, we calculated the percentage of nuclear *vs.* cytoplasmic locations of Nrf2.

Quantification of PDGFRβ+ immunostaining. Every other section, resulting in a total of 10 sections, starting anterior to the lesion and covering the most rostral aspect of the lesion (~2 mm) was analyzed from each rat. As an outcome measure, the rostrocaudal extent and mediolateral coverage (mm) of the PDGFRβ-positive region was measured (see Figure 9A) using ImageJ® software. Shrinkage related to histologic processing of the sections was adjusted as described in [91].

#### 4.2.6. Statistical Analysis

In vitro. Data from the cell culture experiments were analyzed using the linear regression models in R (version 3.5.1) (R Core Team, 2018) [94]. The data are presented as percentages instead of actual values to normalize the batch effect (data are derived from two different batches).

In vivo. In vivo data were analyzed using SPSS for Windows (version 25.0). RT-qPCR data were analyzed in GraphPad Prism 8 (GraphPad Software, USA) for Windows. The Shapiro–Wilks normality test was used to test the distribution of the data. In case of normal distribution, continuous data over the time-points was tested with the parametric repeated measures ANOVA followed by the Tukey HSD post-hoc test (beam walking). Differences between two time-points in the same animals were analyzed using the paired sample *t*-test and differences between groups were analyzed using the independent sample t-test (apnea, righting, Nrf2 cell number). In case of non-normally distributed data, continuous data over the time-points was tested with the non-parametric Friedman test followed by Dunn’s post-hoc analysis (neuroscore). Differences between two time-points in the same animals were analyzed using the Wilcoxon signed-rank test (weight, plasma markers). Differences between the groups at a given time-point was analyzed using the Kruskal–Wallis test followed by the Mann–Whitney *U*-test as a post-hoc test (RT-qPCR data, weight, lesion area, distribution of PDGFRβ in the brain, plasma markers). Correlations between behavioral data or plasma markers with the cortical lesion area were analyzed with Spearman’s rank correlation coefficient. A *p*-value less than 0.05 was considered significant. Data are presented as whisker-plots from minimum to maximum or mean ± standard error of mean (SEM).

## 5. Conclusions

Our in vitro data demonstrated that duotherapy with NAC and SFN dose-dependently decreased neuroinflammation and nitric oxide-mediated neurotoxicity, and increased neuronal survival. In vivo validation revealed that systemic administration of NAC+SFN duotherapy reduced inflammatory plasma biomarker levels in sham-operated experimental controls with craniectomy. The duotherapy resulted in mild mitigation of functional deficits in rats with severe TBI. This study highlights the potential of antioxidant therapy in improving the outcome after a brain injury.

## Figures and Tables

**Table 1:** Mean ± SEM area (mm^2^) and percentage (%) of the lesion area of the total area of the given cytoarchitectonic cortical region. Number of animals with a lesion in a given cortical subfield is in parentheses (n). No difference in lesion area was detected between groups.

	TBI-VEH (n =10)		TBI-DUO (n = 10)	
Area	mm^2^ ± SEM	% (n)	mm^2^ ± SEM	% (n)
PRh	0.35	10% (1/10)	1.02	29% (1/10)
Ect	0.95 ± 0.43	18% (6/10)	1.88 ± 1.08	14% (9/10)
TeA	1.93 ± 0.27	49% (9/10)	6.99 ± 5.01	45% (9/10)
Au1	3.62 ± 0.48	62% (10/10)	6.62 ± 3.51	48% (10/10)
AuD	2.40 ± 0.09	92% (10/10)	9.12 ± 6.88	78% (10/10)
AuV	0.71 ± 0.20	27% (9/10)	2.11 ± 1.43	24% (8/10)
S1	1.19 ± 0.02	97% (10/10)	10.19 ± 8.98	89% (10/10)
S1BF	5.55 ± 0.63	49% (10/10)	6.79 ± 1.61	43% (10/10)
S1DZ	0.12 ± 0.07	7% (3/10)	0.08 ± 0.05	4% (2/10)
S1FL	0.10	1% (1/10)	0	0% (0/10)
S1ULp	1.17 ± 0.23	13% (9/10)	0.84 ± 0.18	8% (8/10)
S2	0.76 ± 0.20	11% (9/10)	0.79 ± 0.25	7% (7/10)
PtPD	0.25 ± 0.09	26% (6/10)	0.32 ± 0.08	33% (10/10)
PtPR	0.50 ± 0.056	67% (10/10)	0.64 ± 0.038	86% (10/10)
V1	0.11 ± 0	5% (2/10)	0.66 ± 0.62	31% (3/10)
V1B	1.45 ± 0.36	28% (8/10)	1.86 ± 0.41	37% (10/10)
V1M	1.21 ± 0.52	21% (5/10)	0.92 ± 0.35	16% (6/10)
V2L	4.46 ± 0.40	69% (10/10)	5.21 ± 0.30	80% (10/10)
V2ML	0.29 ± 0.17	9% (2/10)	0.19 ± 0.05	6% (3/10)
V2MM	0.75	18% (1/10)	0.32 ± 0.09	8% (2/10)
RSD	1.02	10% (1/10)	0	0% (0/10)
Total lesion area	24.65 ± 3.21	5% (10/10)	24.87 ± 3.24	5% (10/10)

Abbreviations: Au1, primary auditory cortex; AuD, secondary auditory cortex, dorsal area; AuV, secondary auditory cortex, ventral area; Ect, ectorhinal cortex; PRh, perirhinal cortex; PtPD, parietal cortex, posterior area, dorsal part; PtPR, parietal cortex, posterior area, rostral part; RSD, retrosplenial dysgranular cortex; S1, primary somatosensory cortex; S1BF, primary somatosensory cortex, barrel field; S1DZ, primary somatosensory cortex, dysgranular zone; S1FL, primary somatosensory cortex, forelimb region; S1ULp, primary somatosensory cortex, upper lip region; S2, secondary somatosensory cortex; TBI, traumatic brain injury; TeA, temporal associatin cortex; V1, primary visual cortex; V1B, primary visual cortex, binocular area; V1M, primary visual cortex, monocular area; V2L, secondary visual cortex, lateral area; V2ML, secondary visual cortex, mediolateral area; V2MM, secondary visual cortex, mediomedial area.

## Supplementary Materials

The following are available online at https://www.mdpi.com/article/10.3390/ijms221910555/s1.
