# Supplementary material for: Targeting Oxidative Stress with Antioxidant Duotherapy after Experimental Traumatic Brain Injury
Source: Int J Mol Sci. 2021 Sep 29;22(19):10555. doi: 10.3390/ijms221910555 (PMC8508668; doi:10.3390/ijms221910555)
Supplement: Supplementary file 1 [file ijms-22-10555-s001.zip › ijms-1377272-supplementary.pdf]

Targeting oxidative stress with antioxidant duotherapy after experimental traumatic brain injury

Jenni Kyyriäinen, Natallie Kajevu, Ivette Bañuelos, Leonardo Lara, Anssi Lipponen, Silvia Balosso, Elina Hämäläinen, Shalini Das Gupta, Noora Puhakka, Teemu Natunen, Teresa Ravizza, Annamaria Vezzani, Mikko Hiltunen, Asla Pitkänen

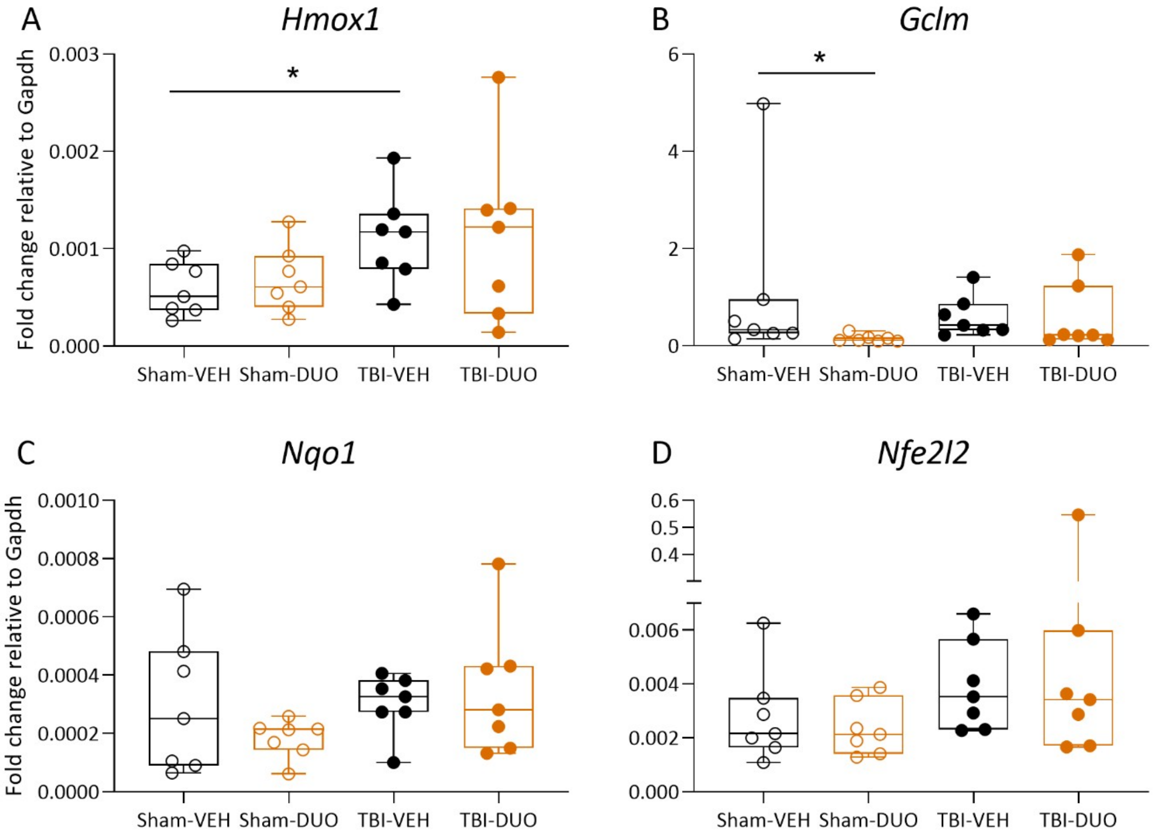

Figure 1. Expression of Nrf2 target genes in blood cells on day (D) 6 post-TBI. **(A)** *Hmox1* gene expression was higher in the TBI-VEH group than in the Sham-VEH group ($p<0.05$). No injury effect was detected between the Sham-DUO and TBI-DUO groups ($p>0.05$). **(B)** *Gclm* gene expression was lower in the Sham-DUO group than in the Sham-VEH group ($p<0.05$). No treatment effect was detected in the TBI group (TBI-VEH vs. TBI-DUO, $p>0.05$). **(C)** *Nqo1* and **(D)** *Nfe2l2* gene expression did not differ between groups ($p>0.05$). Gene expression was calculated relative to expression of the reference gene (*Gapdh*, glyceraldehyde 3-phosphate dehydrogenase) on D6 post-TBI Abbreviations: D, day; DUO, duotherapy treated with N-acetylcysteine and sulforaphane; *Gapdh*, glyceraldehyde 3-phosphate dehydrogenase; *Gclm*, glutamate-cysteine ligase modifier subunit; *Hmox1*, heme oxygenase 1; *Nfe2l2*, nuclear factor erythroid 2 like 2; *Nqo1*, NAD(P)H quinone oxidoreductase 1; TBI, traumatic brain injury; VEH, vehicle. Statistical significances: * $p<0.05$ (Kruskal-Wallis followed

by Mann-Whitney *U*-test). Data are presented as whisker-plots with mean and minimum/maximum. Note different scales in the y-axis.

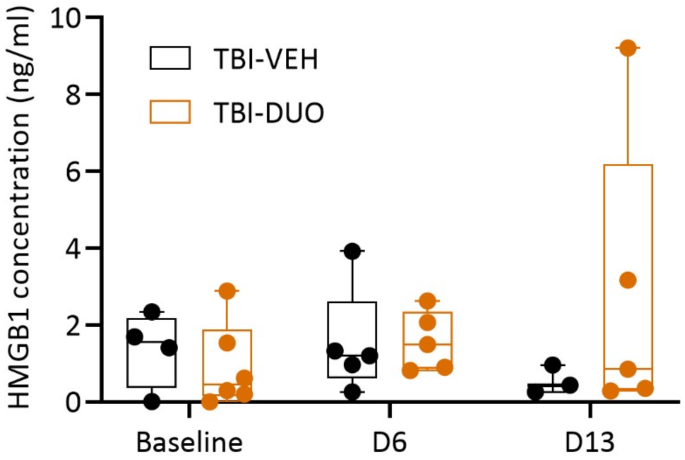

Figure S2. Plasma high-mobility group box 1 (HMGB1) concentration (ng/ml) at baseline and on day (D) 6 and D13 post-TBI. No difference was detected between the time-points ($p>0.05$, Wilcoxon signed-rank test) or between TBI-VEH and TBI-DUO groups at any time-point ($p>0.05$, Mann-Whitney *U*-test). Data are presented as whisker-plots with mean and minimum/maximum. Abbreviations: D, day; DUO, duotherapy treated with N-acetylcysteine and sulforaphane; HMGB1, high-mobility group box 1; TBI, traumatic brain injury; VEH, vehicle.

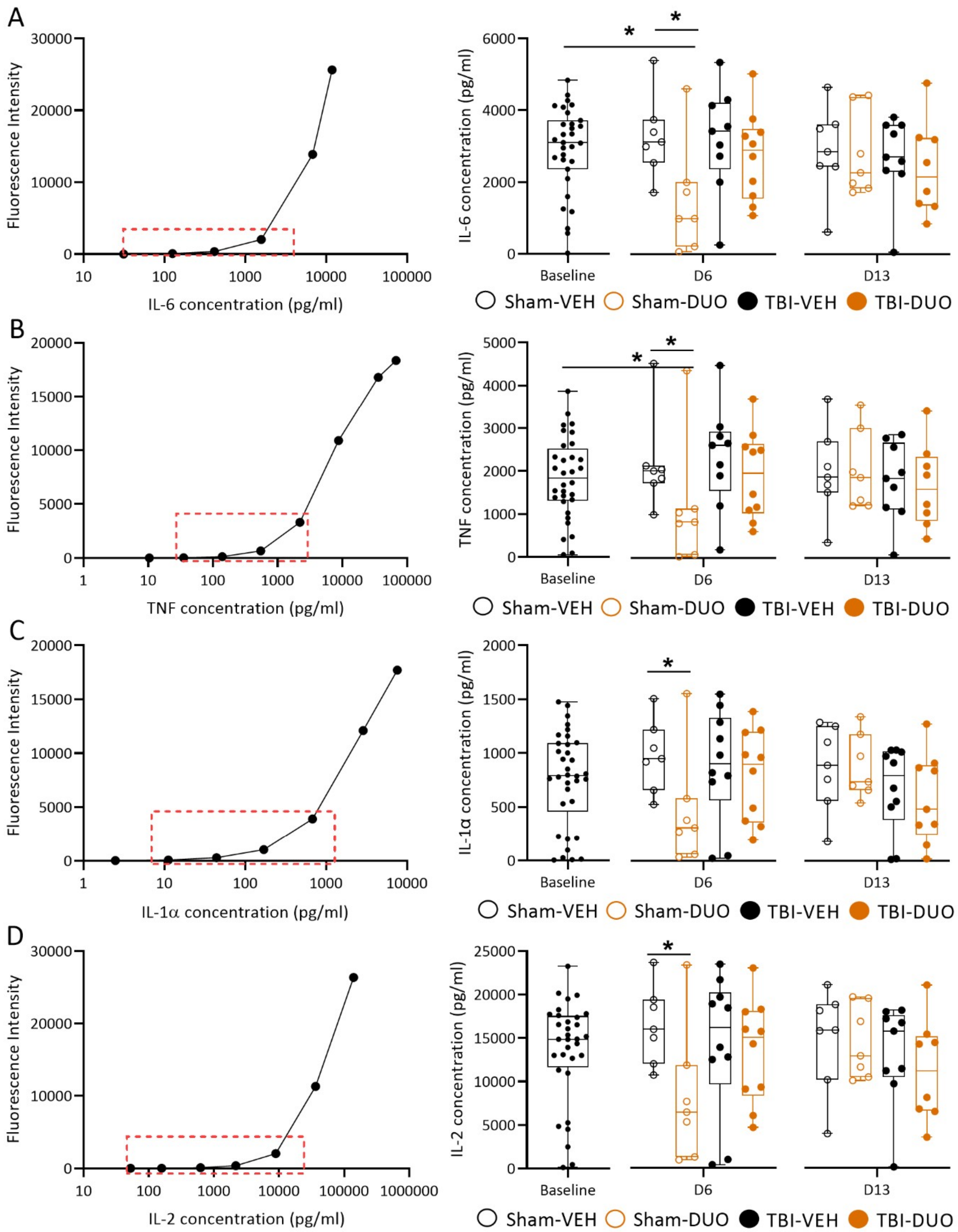
A
Fluorescence Intensity
IL-6 concentration (pg/ml)
Baseline
D6
D13
Sham-VEH
Sham-DUO
TBI-VEH
TBI-DUO
B
TNF concentration (pg/ml)
C
IL-1α concentration (pg/ml)
D
IL-2 concentration (pg/ml)

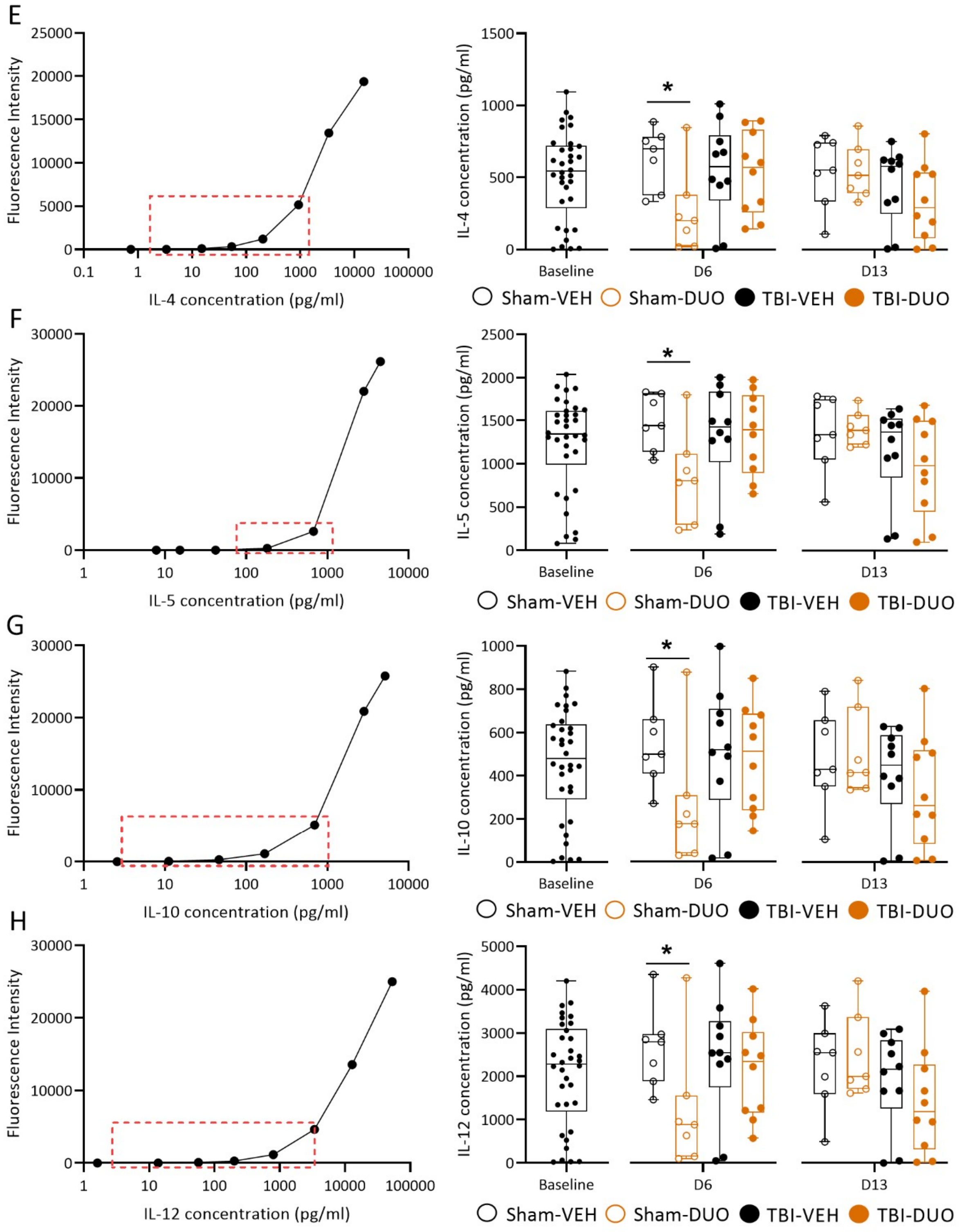
E
Fluorescence Intensity
IL-4 concentration (pg/ml)
IL-4 concentration (pg/ml)
Baseline
D6
D13
Sham-VEH
Sham-DUO
TBI-VEH
TBI-DUO
F
IL-5 concentration (pg/ml)
G
IL-10 concentration (pg/ml)
H
IL-12 concentration (pg/ml)

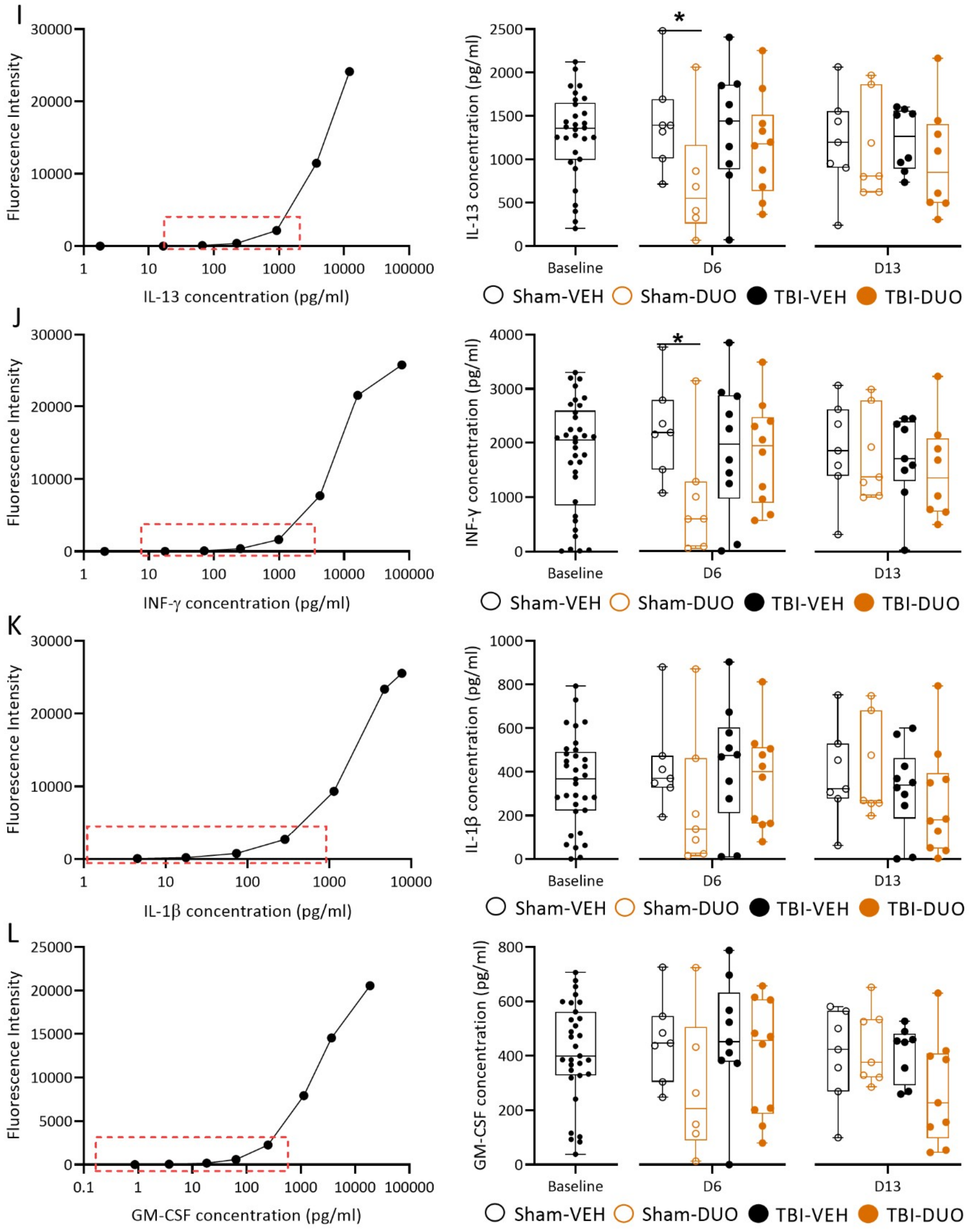

FigureS3. Plasma cytokine concentrations (pg/ml) at baseline, day (D) 6, and D13 post-TBI. Panels on the left show the standard curves separately for each cytokine with the detection range indicated by a red dashed box. Whisker-plots with mean and minimum/maximum on the right show the concentration of each cytokine at baseline (n = 34, including all animals) and on D6 and D13 post-TBI in the 4 treatment groups. Overall, no injury effect on cytokine levels was detected on D6 or D13

when the TBI-VEH group was compared with the Sham-VEH group or the TBI-DUO group was compared with the Sham-DUO group ($p>0.05$). Also, no treatment effect on cytokine levels was detected in the TBI-DUO group on D6 or D13 (TBI-DUO vs TBI-VEH, all $p>0.05$). **(A)** On D6, however, IL-6 levels were decreased in the Sham-DUO group compared with baseline or with the Sham-VEH group (both, $p<0.05$). **(B)** On D6, TNF levels were decreased in the Sham-DUO group compared with baseline or with the Sham-VEH group (both, $p<0.05$). Furthermore, a decrease was detected in the Sham-DUO group compared with the Sham-VEH group in **(C)** IL-1α, **(D)** IL-2, **(E)** IL-4, **(F)** IL-5, **(G)** IL-10, **(H)** IL-12, **(I)** IL-13, and **(J)** INF-γ (all, $p<0.05$). No difference was detected in **(K)** IL-1β. **(L)** GM-CSF levels. Abbreviations: D, day; DUO, duotherapy treated with N-acetylcysteine and sulforaphane; GM-CSF, granulocyte-macrophage colony-stimulating factor; INF-γ interferon gamma; IL-1α, interleukin 1 alpha; IL-1β, interleukin 1 beta; IL-2, interleukin 2; IL-4, interleukin 4; IL-5, interleukin 5; IL-6, interleukin 6; IL-10, interleukin 10; IL-12, interleukin 12; IL-13, interleukin 13; TBI, traumatic brain injury; TNF, tumor necrosis factor alpha; VEH, vehicle. Statistical significances: * $p<0.05$, Mann-Whitney *U* test. Data are presented as whisker-plots with mean and minimum/maximum.
